# Author Correction: Disrupting TSLP–TSLP receptor interactions via putative small molecule inhibitors yields a novel and efficient treatment option for atopic diseases

**DOI:** 10.1038/s44321-024-00165-4

**Published:** 2024-11-07

**Authors:** Partho Protim Adhikary, Temilolu Idowu, Zheng Tan, Christopher Hoang, Selina Shanta, Malti Dumbani, Leah Mappalakayil, Bhuwan Awasthi, Marcel Bermudez, January Weiner, Dieter Beule, Gerhard Wolber, Brent D G Page, Sarah Hedtrich

**Affiliations:** 1 Faculty of Pharmaceutical Sciences, The University of British Columbia, Vancouver, BC Canada; 2 Institute of Pharmacy, Freie Universität of Berlin, Berlin, Germany; 3 Institute of Pharmaceutical and Medicinal Chemistry, Westfälische Wilhelms-Universität Münster, Münster, Germany; 4 Berlin Institute of Health at Charité - Universitätsmedizin Berlin, Germany Charité - Universitätsmedizin Berlin, Berlin, Germany; 5 Department of Infectious Diseases and Respiratory Medicine, Charité—Universitätsmedizin Berlin, corporate member of Freie Universität Berlin and Humboldt Universität zu Berlin, Berlin, Germany; 6 Max-Delbrück Center for Molecular Medicine in the Helmholtz Association (MDC), 13125 Berlin, Germany

## Abstract

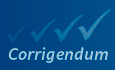

**Correction to:** *EMBO Molecular Medicine* (2024) 16:1630–1656. 10.1038/s44321-024-00085-3 | Published online 14 June 2024

**Figure 2. Original.**

**Figure:**
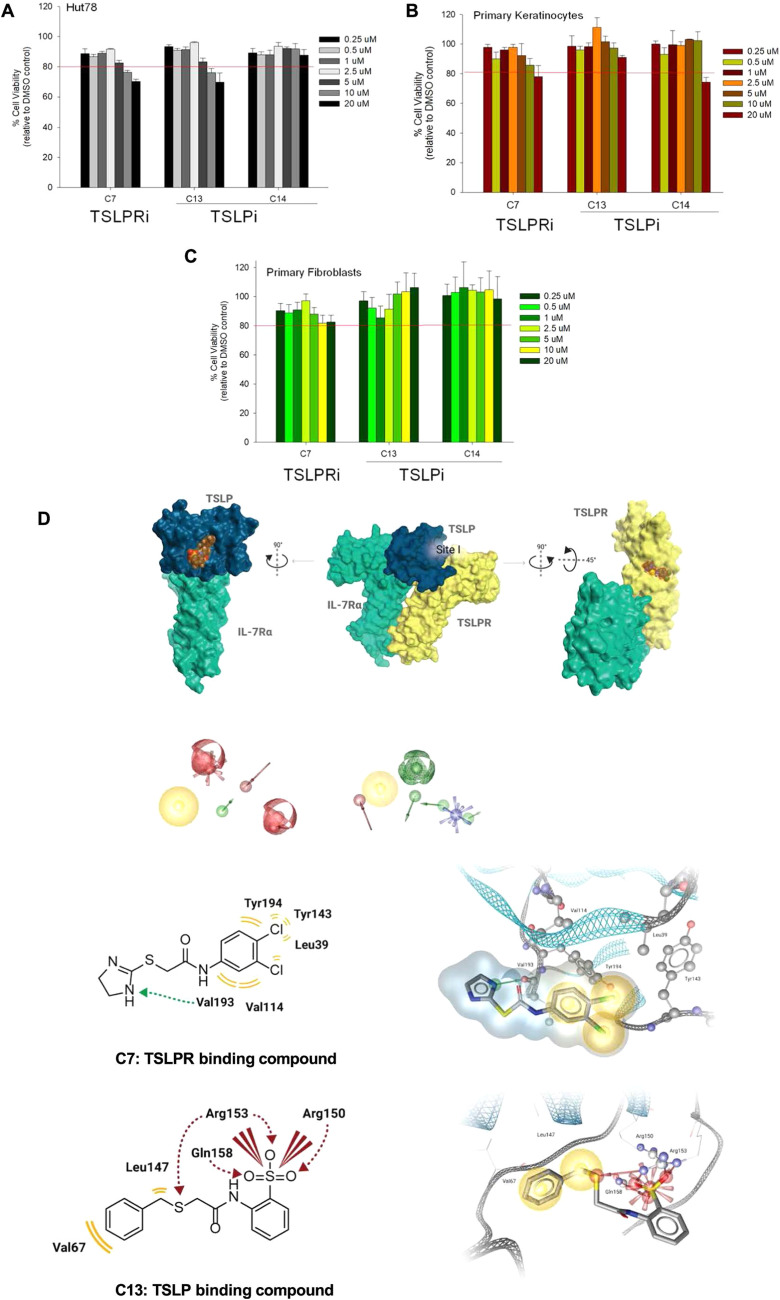

**Figure 2. Corrected.**

**Figure:**
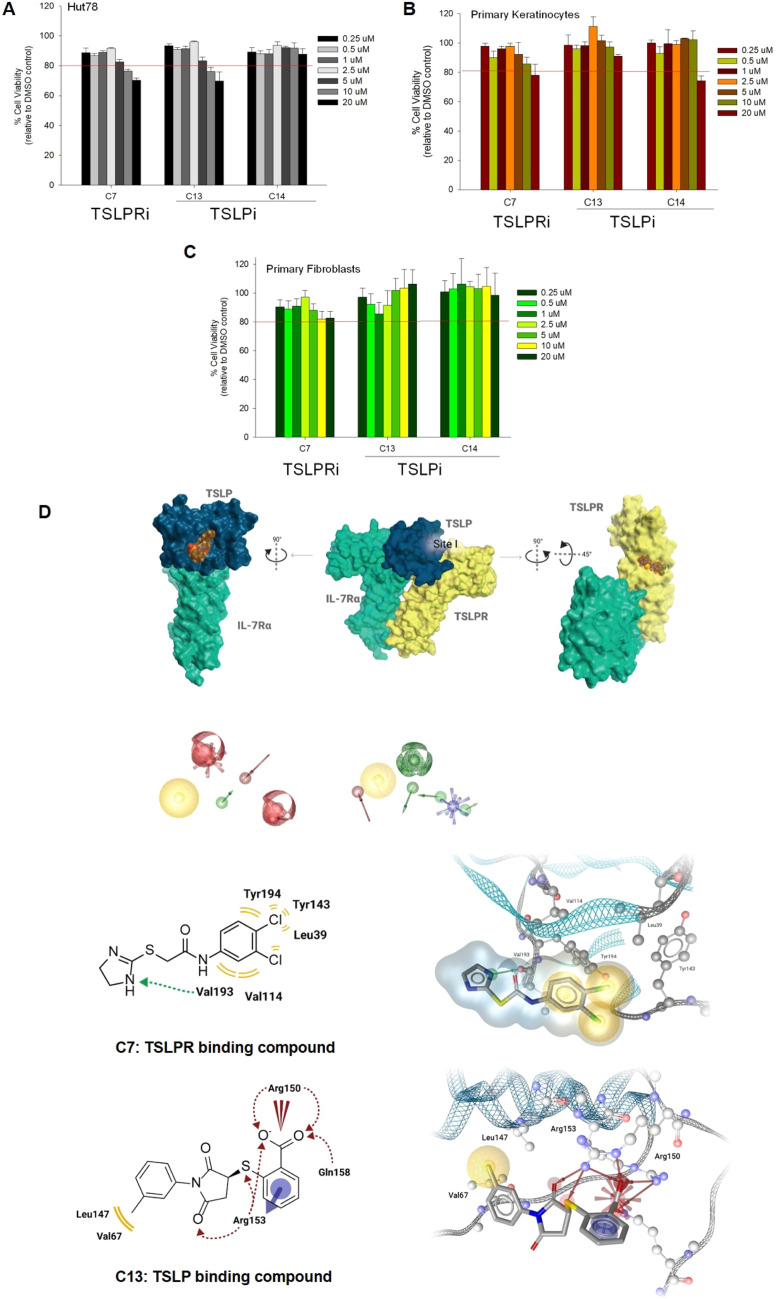

Figure 2D is corrected after the journal was informed there was an error in the figure. The authors confirmed the error and have produced the correct 2D and 3D structure.

Author statement: In Fig. 2D (both the 2D and 3D structure), the wrong structure of C13 has been displayed. The correct structure is displayed in the other figures (Figs. 1 and 3). We provide the corrected Figure 2D and apologize for the inconvenience.

The correction does not affect the conclusion of the manuscript.

The original article has been corrected.

