# Supplementary material for: Disrupting TSLP–TSLP receptor interactions via putative small molecule inhibitors yields a novel and efficient treatment option for atopic diseases
Source: EMBO Mol Med. 2024 Jun 14;16(7):9. doi: 10.1038/s44321-024-00085-3 (PMC11250841; doi:10.1038/s44321-024-00085-3)
Supplement: Supplementary file 1 — Appendix [file 44321_2024_85_MOESM1_ESM.pdf]

Appendix

Disrupting TSLP – TSLP receptor interaction via small molecule inhibitors yields a novel and efficient treatment option for atopic diseases

Partho Protim Adhikary[1], Temi Idowu[1], Zheng Tan[1], Christopher Hoang[1], Selina Shanta[1], Malti Dumbani[2], Leah Mappalakayil[1], Bhuwan Awasthi[1], Marcel Bermudez[2,3], January Weiner[4], Dieter Beule[4], Gerhard Wolber[2], Brent D. G Page*[1], Sarah Hedtrich*[1,4,5,6]

[1] Faculty of Pharmaceutical Sciences, The University of British Columbia, Vancouver, BC, Canada

[2] Institute of Pharmacy, Freie Universität of Berlin, Berlin, Germany

[3] Institute of Pharmaceutical and Medicinal Chemistry, Westfälische Wilhelms-Universität Münster

[4] Berlin Institute of Health at Charité - Universitätsmedizin Berlin, Germany Charité – Universitätsmedizin Berlin, Germany

[5] Department of Infectious Diseases and Respiratory Medicine, Charité - Universitätsmedizin Berlin, corporate member of Freie Universität Berlin and Humboldt Universität zu Berlin, Germany.

[6] Max-Delbrück Center for Molecular Medicine in the Helmholtz Association (MDC), 13125 Berlin, Germany

***Corresponding Author:** Dr. Sarah Hedtrich, Berlin Institute of Health @ Charité Universitätsmedizin, Berlin, Germany· Lindenberger Weg 80, 13125 Berlin, Germany. Email: sarah.hedtrich@bih-charite.de, phone: +4930450541015. Dr. Brent Page, Faculty of Pharmaceutical Sciences, The University of British Columbia, 2405 Wesbrook Mall, Vancouver, V6T1Z3, Canada. Email: brent.page@ubc.ca

Table of Content

Appendix Tables	Page 3
Appendix Figures	Page 12
Appendix Synthetic Protocols & Characterization	Page 18
Appendix References	Page 49

1. Appendix Tables

Appendix Table S1. Compound screening from NCI database. Chemical structures are shown for compounds that reduced either IL4 or IL13 expression to ≤ 25 % of untreated controls.

NCI ID	IL-13 (% of Expression)	STDEV	IL-4 (% of Expression)	STDEV
13338	157.9586685	52.02470483	39.92447717	34.59033482
13339	101.7192891	19.89239106	46.87868513	13.93669869
13342	82.91757193	40.04528331	48.58906566	17.05027517
13345	98.28209749	30.0078599	51.41325196	13.69454416
13350	11.53711767	4.197424112	44.54472859	29.06898047
13363	3.528434332	4.129453496	35.9373194	42.22981949
13367	137.9846965	43.67141571	67.06041373	11.78688859
34270	118.4484733	30.29911612	50.51714577	13.93292014
34649	93.54965801	24.14431646	52.82968587	17.38101169
36738	122.6043204	35.88973884	37.67275194	15.21448067
49433	92.88961751	25.2966316	34.15179688	14.16944917
49435	33.77542861	6.687404024	34.5609282	15.2364769
52658	89.74969935	15.93907886	91.1574161	2.916973451
52645	92.7890318	43.38224945	88.18708855	68.54201857
151065	8.402860298	27.34897833	23.35265802	10.82766685
111074	104.0524403	26.85317751	88.28954331	8.750198507
39562	24.13587532	21.27147534	36.05324779	37.91704564
164991	29.14183465	17.73681559	67.80468639	37.91704564
159043	38.97496904	24.31022683	68.11197896	7.292072704
404246	50.05959326	6.77556708	58.58655455	8.750198507
190460	146.9606631	16.24930569	111.2325257	37.91704564
159045	49.16567196	3.038751495	58.68893761	24.79174787
193385	68.65315648	91.16340434	47.0126086	24.79174787
190504	63.28962863	19.24101477	98.22464334	51.0423434
193387	80.27413348	18.12465284	68.52165459	36.45891983
150534	50.59594605	9.116361921	47.31990117	43.75027069
39561	18.77234747	29.891929	58.79139237	49.58349575
193365	85.99522986	15.19386491	98.63431897	59.7925419
193362	75.0893899	30.38783726	73.43804893	4.375099254
634641	23.7783068	33.42658875	38.61382802	24.79174787
193391	34.14779398	21.27147534	67.70230333	51.0423434
161058	44.33849689	48.62045366	83.68044154	16.04154937
190461	46.48390803	12.15511342	73.95017931	2.916973451
160947	30.03575596	36.46534025	81.01740655	27.70872132
150558	32.35995136	3.038751495	75.99862916	35.00007218
148169	32.71751988	393.2198582	46.60293297	1.458125803
193390	38.43861626	3.038751495	26.32298557	16.04154937
99249	53.6352785	18.26431036	109.4912967	7.292072704
379408	172.3480282	24.31022683	138.6821551	192.5014798
202079	99.94040227	24.06362426	111.7445844	13.12529776
211870	80.64178645	15.730825	71.19898731	29.31412188
204173	57.03644224	12.4066842	37.79935907	18.50327134
212419	50.94735905	16.09971657	39.80922075	31.17988679
211871	69.71188212	19.87038157	57.24903622	9.79342792
246985	83.72692518	14.98444433	62.36500229	9.443505109
211926	77.90169901	18.26825696	57.86520368	62.27890906
211974	61.88741022	11.78823889	49.52380238	12.60016173
211901	69.89455462	26.88438531	50.47594745	61.35999412
211980	67.93590115	25.31905515	67.9422809	9.002425935
211861	54.67184664	22.48868844	56.42823013	11.15195178
204296	106.3884615	27.00511479	208.5645525	123.7300595
15237	92.73700306	78.57142857	38.37620383	26.72077544
33719	12.60512232	51.78571429	0.333236931	20.36363636
37312	70.44629205	24.10714286	74.44629205	34.10714286
37314	88.31708716	175.8928571	81.31708716	175.8928571
148169	84.1838685	14.28571429	84.1838685	14.28571429
150534	72.64430428	54.46428571	72.64430428	54.46428571
151097	8.041857798	29.46428571	0.65908224	29.02164502
152171	10.02484709	8.928571429	1.706532506	30.92640693
152202	11.76892202	0.892857143	4.522735585	56.9004329
153391	82.86983945	25.89285714	74.86983945	35.89285714
159043	41.65711009	16.07142857	41.65711009	16.07142857
159045	44.52408257	8.928571429	44.52408257	8.928571429
163534	74.36448777	88.39285714	74.36448777	88.39285714
164999	79.93119266	14.28571429	79.93119266	14.28571429
165003	56.97152141	41.96428571	8.99670475	112.6580087
165012	72.83543578	79.46428571	72.83543578	79.46428571
176306	35.87538226	48.02816306	1.112269821	17.59307359
176382	73.67163609	64.28571429	73.67163609	64.28571429
17637	57.80772171	32.14285714	57.80772171	32.14285714
190503	65.3574159	180.3571429	65.3574159	180.3571429
190525	83.10875382	50.89285714	83.10875382	50.89285714
193363	74.22113914	12.67857143	74.22113914	12.67857143
201503	45.57530581	14.28571429	6.047823344	157.8528139
201555	79.90730122	38.39285714	79.90730122	38.39285714
202084	83.992737	46.42857143	83.992737	46.42857143
209955	80.67182722	19.82142857	80.67182722	19.82142857
246087	83.25210245	47.32142857	83.25210245	47.32142857
332174	102.078555	31.25	102.078555	31.25
338570	51.54816514	28.57142857	6.192856272	17.9605302
379408	57.09097859	38.92857143	47.09097859	18.92857143
19846	63.78058104	26.07142857	68.78058104	31.07142857
636345	59.64736239	27.67857143	39.64736239	27.67857143
636379	95.5800841	25.53571429	55.56503718	10.88422442

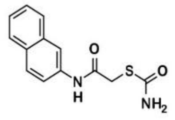

NSC13363
%IL13 = 3.5
%IL4 = 36

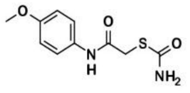

NSC13350
%IL13 = 12
%IL4 = 45

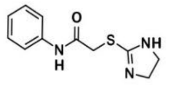

NSC33719
%IL13 = 13
%IL4 = 0.33

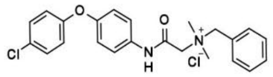

NSC151097
%IL13 = 8.0
%IL4 = 0.66

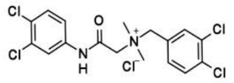

NSC151065
%IL13 = 8.4
%IL4 = 23

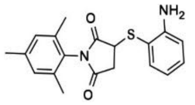

NSC176306
%IL13 = 36
%IL4 = 1.1

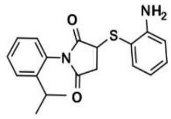

NSC176306
%IL13 = 52
%IL4 = 6.2

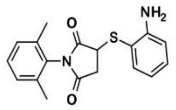

NSC165003
%IL13 = 57
%IL4 = 9.0

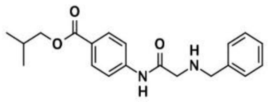

NSC39561
%IL13 = 19
%IL4 = 59

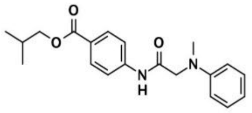

NSC39562
%IL13 = 24
%IL4 = 36

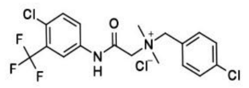

NSC152171
%IL13 = 10
%IL4 = 31

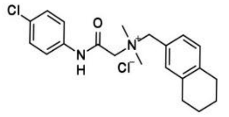

NSC152202
%IL13 = 12
%IL4 = 4.5

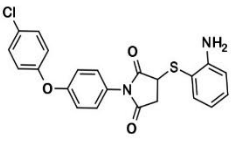

NSC201503
%IL13 = 45
%IL4 = 6.0

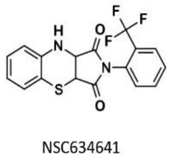

NSC634641
%IL13 = 24
%IL4 = 39

Appendix Table S2: Fractional Atomic Coordinates (×10[4]) and Equivalent Isotropic Displacement Parameters (Å^2×10^3) for **BP79**. U_{eq} is defined as 1/3 of the trace of the orthogonalised U_{ij}.

**Atom**	**x**	**y**	**z**	$\boldsymbol{U_{eq}}$
Cl1	8633.8(13)	3855.0(2)	9204.6(4)	16.16(11)
Cl2	7295.3(13)	5201.8(2)	8782.1(4)	16.11(11)
O1	5473(4)	2628.6(6)	5873.5(12)	20.5(3)
O2	84(4)	3871.7(5)	3402.0(11)	20.0(3)
N1	3304(4)	3506.7(6)	5252.3(13)	12.8(3)
N2	-2125(5)	3452.7(7)	1838.7(14)	16.5(3)
C1	4302(5)	3877.5(7)	6135.7(14)	11.4(3)
C2	5862(5)	3692.2(7)	7142.6(15)	12.7(3)
C3	6732(5)	4102.8(7)	7956.1(14)	11.1(3)
C4	6090(5)	4692.7(7)	7779.4(14)	11.8(3)
C5	4493(5)	4875.9(7)	6781.6(15)	13.4(3)
C6	3595(5)	4471.8(7)	5968.4(15)	12.9(3)
C7	3904(5)	2931.0(7)	5154.2(15)	12.9(3)
C8	2620(5)	2645.9(7)	4096.3(15)	13.8(3)
C9	918(5)	2834.1(7)	3160.8(15)	13.3(3)
C10	-388(5)	3428.5(7)	2826.1(15)	12.6(3)

Appendix Table S3: Anisotropic Displacement Parameters (×10[4]) for **BP79**. The anisotropic displacement factor exponent takes the form: -2□$^2[h^2a^{*2} \times U_{11} + ... +2hka^* \times b^* \times U_{12}]$

**Atom**	$\boldsymbol{U_{11}}$	$\boldsymbol{U_{22}}$	$\boldsymbol{U_{33}}$	$\boldsymbol{U_{23}}$	$\boldsymbol{U_{13}}$	$\boldsymbol{U_{12}}$
Cl1	20.4(2)	17.65(19)	10.0(2)	1.43(14)	-3.38(16)	1.65(16)
Cl2	20.3(2)	15.04(18)	12.7(2)	-3.54(15)	-1.67(16)	-1.13(15)
O1	31.4(9)	14.3(6)	15.1(7)	2.0(5)	-6.3(6)	4.4(5)
O2	33.6(9)	12.2(5)	13.2(6)	-2.1(5)	-7.3(6)	2.9(5)
N1	16.7(8)	11.5(6)	9.9(7)	0.7(5)	-2.6(6)	1.0(5)
N2	23.8(9)	12.6(6)	12.6(8)	-0.1(6)	-4.3(6)	-1.3(6)
C1	12.1(8)	12.4(7)	9.8(8)	0.0(6)	1.0(6)	-1.2(6)
C2	14.9(9)	12.7(7)	10.5(8)	1.0(6)	0.5(6)	0.9(6)
C3	10.4(8)	15.1(7)	7.6(8)	1.4(6)	-0.6(6)	0.8(6)
C4	12.6(8)	13.0(7)	9.8(8)	-2.5(6)	0.4(6)	-1.0(6)
C5	16.8(9)	12.0(7)	11.6(8)	0.2(6)	1.2(7)	1.0(6)
C6	13.6(9)	13.6(7)	11.3(8)	1.3(6)	-1.1(6)	1.3(6)
C7	14.4(9)	12.5(7)	11.7(8)	0.8(6)	1.3(6)	-0.6(6)
C8	16.4(9)	9.8(7)	15.3(9)	-0.5(6)	0.4(7)	-0.5(6)
C9	15.2(9)	11.3(7)	13.4(8)	-2.0(6)	-0.6(7)	-0.4(6)
C10	13.1(9)	12.8(7)	11.7(8)	0.7(6)	-0.3(6)	-1.4(6)

Appendix Table S4: Bond Lengths in Å for **BP79**.

**Atom**	**Atom**	**Length/Å**
Cl1	C3	1.7339(17)
Cl2	C4	1.7286(17)
O1	C7	1.236(2)
O2	C10	1.241(2)
N1	C1	1.403(2)
N1	C7	1.344(2)
N2	C10	1.332(2)
C1	C2	1.391(2)
C1	C6	1.400(2)
C2	C3	1.390(2)
C3	C4	1.387(2)
C4	C5	1.386(2)
C5	C6	1.382(2)
C7	C8	1.497(2)
C8	C9	1.341(3)
C9	C10	1.495(2)

Appendix Table S5: Bond Angles in ° for **BP79**.

Atom	Atom	Atom	Angle/°
C7	N1	C1	128.63(16)
C2	C1	N1	124.62(15)
C2	C1	C6	119.36(16)
C6	C1	N1	116.02(15)
C3	C2	C1	119.23(15)
C2	C3	Cl1	117.93(13)
C4	C3	Cl1	120.85(13)
C4	C3	C2	121.22(16)
C3	C4	Cl2	120.87(14)
C5	C4	Cl2	119.63(13)
C5	C4	C3	119.49(15)
C6	C5	C4	119.85(16)
C5	C6	C1	120.83(16)
O1	C7	N1	124.14(17)
O1	C7	C8	118.58(15)
N1	C7	C8	117.28(16)
C9	C8	C7	134.51(16)
C8	C9	C10	130.84(16)
O2	C10	N2	121.40(16)
O2	C10	C9	123.99(16)
N2	C10	C9	114.60(15)

Appendix Table S6: Torsion Angles in ° for **BP79**.

Atom	Atom	Atom	Atom	Angle/°
Cl1	C3	C4	Cl2	2.0(2)
Cl1	C3	C4	C5	-178.57(14)
Cl2	C4	C5	C6	178.63(15)
O1	C7	C8	C9	-179.0(2)
N1	C1	C2	C3	-179.98(17)
N1	C1	C6	C5	-179.54(17)
N1	C7	C8	C9	0.2(3)
C1	N1	C7	O1	0.9(3)
C1	N1	C7	C8	-178.25(17)
C1	C2	C3	Cl1	179.43(14)
C1	C2	C3	C4	-0.3(3)
C2	C1	C6	C5	1.3(3)
C2	C3	C4	Cl2	-178.21(14)
C2	C3	C4	C5	1.2(3)
C3	C4	C5	C6	-0.8(3)
C4	C5	C6	C1	-0.5(3)
C6	C1	C2	C3	-0.9(3)
C7	N1	C1	C2	-6.4(3)
C7	N1	C1	C6	174.49(18)
C7	C8	C9	C10	-0.3(4)
C8	C9	C10	O2	3.0(3)
C8	C9	C10	N2	-177.3(2)

Appendix Table S7: Hydrogen Fractional Atomic Coordinates ($\times 10^4$) and Equivalent Isotropic Displacement Parameters ($Å^2 \times 10^3$) for **BP79**. U_{eq} is defined as 1/3 of the trace of the orthogonalised U_{ij}.

Atom	x	y	z	U_{eq}
H2	6326.93	3289.71	7272.84	15
H5	4016.36	5278.5	6656.66	16
H6	2484.43	4598.98	5287.46	15
H8	3126.12	2239.61	4084.96	17
H9	465.8	2540.05	2619.94	16
H1	2240(80)	3661(12)	4670(20)	34(7)
H2A	-2940(80)	3782(12)	1650(20)	35(8)
H2B	-2610(70)	3167(12)	1480(20)	30(7)

Appendix Table S8. Top 20 genes with at least 2-fold change differentially expressed between diseased skin, treated and diseased skin, untreated. Log_2 fold change greater than zero indicates higher expression in the treated tissue.

ENSEMBLID	Symbol	Description	log2FC	P value	FDR
ENSG00000071991	CDH19	cadherin 19	20.4	< 2e-16	2.1e-15
ENSG00000108379	WNT3	Wnt family member 3	<2e-16	6.2e-14	1.2e-10
ENSG00000113108	APBB3	amyloid beta precursor protein binding family B member 3	<2e-16	3.3e-13	4.5e-10
ENSG00000113946	CLDN16	claudin 16	<2e-16	< 2e-16	2.8e-13
ENSG00000114315	HES1	hes family bHLH transcription factor 1	<2e-16	5.0e-15	1.3e-11
ENSG00000121931	LRIF1	ligand dependent nuclear receptor interacting factor 1	2.4	1.2e-15	3.6e-12
ENSG00000125686	MED1	mediator complex subunit 1	1.5	2.6e-16	9.1e-13
ENSG00000133247	KMT5C	lysine methyltransferase 5C	<2e-16	7.6e-13	8.0e-10
ENSG00000136870	ZNF189	zinc finger protein 189	3.0	< 2e-16	< 2e-16
ENSG00000158863	FAM160B2	family with sequence similarity 160 member B2	<2e-16	1.4e-11	1.0e-08
ENSG00000166189	HPS6	HPS6 biogenesis of lysosomal organelles complex 2 subunit 3	3.7	< 2e-16	< 2e-16
ENSG00000170477	KRT4	keratin 4	3.9	6.5e-13	7.2e-10
ENSG00000171492	LRRC8D	leucine rich repeat containing 8 VRAC subunit D	1.8	4.0e-13	5.0e-10
ENSG00000177352	CCDC71	coiled-coil domain containing 71	2.7	7.8e-12	6.0e-09

ENSEMBLID	Symbol	Description	log2FC	P value	FDR
ENSG00000180543	TSPYL5	TSPY like 5	4.0	4.0e-12	3.3e-09
ENSG00000185085	INTS5	integrator complex subunit 5	1.9	3.4e-12	3.0e-09
ENSG00000205730	ITPRIPL2	ITPRIP like 2	1.3	2.1e-13	3.1e-10
ENSG00000241484	ARHGAP8	Rho GTPase activating protein 8	<2e-16	7.5e-14	1.3e-10
ENSG00000253797	UTP14C	UTP14C small subunit processome component	1.5	6.3e-13	7.2e-10
ENSG00000278318	ZNF229	zinc finger protein 229	3.0	1.3e-12	1.3e-09

Appendix Table S9: Top 20 genes with at least 2-fold change differentially expressed between 3D lung models co-cultivated with untreated and BP79-treated skin disease models. Log_2 fold change greater than zero indicates higher expression in the treated samples.

ENSEMBLID	Symbol	Description	log2FC	P value	FDR
ENSG00000143520	FLG2	filaggrin family member 2	14.2	1.9e-08	4e-04
ENSG00000130821	SLC6A8	solute carrier family 6 member 8	1.2	0.00020	1e+00
ENSG00000196154	S100A4	S100 calcium binding protein A4	1.1	0.00072	1e+00
ENSG00000180251	SLC9A4	solute carrier family 9 member A4	1.7	0.00099	1e+00
ENSG00000180861	LINC01559	long intergenic non-protein coding RNA 1559	2.3	0.00103	1e+00
ENSG00000070669	ASNS	asparagine synthetase (glutamine-hydrolyzing)	-1.4	0.00104	1e+00
ENSG00000100078	PLA2G3	phospholipase A2 group III	4.0	0.00217	1e+00
ENSG00000207554	MIR647	microRNA 647	1.7	0.00242	1e+00
ENSG00000167771	RCOR2	REST corepressor 2	1.7	0.00267	1e+00
ENSG00000181333	HEPHL1	hephaestin like 1	1.3	0.00283	1e+00
ENSG00000105672	ETV2	ETS variant transcription factor 2	3.7	0.00303	1e+00
ENSG00000135773	CAPN9	calpain 9	1.2	0.00310	1e+00
ENSG00000198734	F5	coagulation factor V	2.1	0.00318	1e+00
ENSG00000101448	EPPIN	epididymal peptidase inhibitor	1.9	0.00379	1e+00
ENSG00000092621	PHGDH	phosphoglycerate dehydrogenase	-1.2	0.00385	1e+00
ENSG00000136859	ANGPTL2	angiopoietin like 2	3.2	0.00409	1e+00
ENSG00000079308	TNS1	tensin 1	1.6	0.00469	1e+00
ENSG00000166323	C11orf65	chromosome 11 open reading frame 65	-1.1	0.00499	1e+00
ENSG00000225833	GS1-594A7.3	uncharacterized LOC104798195	1.2	0.00518	1e+00
ENSG00000130701	RBBP8NL	RBBP8 N-terminal like	1.3	0.00519	1e+00

Appendix Table S10: Exact p values and statistical test information of main Figures 1-8 and Appendix Fig. S1.

Fig. 1C (IL-4_Hut78_screening)
Statistics: Unpaired t-test

C2				C6		
5 µM	10 µM	20 µM		5 µM	10 µM	20 µM
p=0.0015	p=0.0004	p=0.0000003592		p=0.0049	p=0.0004	p=0.0000000007

C7				C13		
5 µM	10 µM	20 µM		5 µM	10 µM	20 µM
p=0.0000000000	p=0.0000000000	p=0.0000000000		p=0.003	p=0.0000010115	p=0.0000007693

Fig. 1D (IL-13_Hut78_screening)
Statistics: Unpaired t-test

C2				C6		
5 µM	10 µM	20 µM		5 µM	10 µM	20 µM
p= 0.0013	p=0.0000209993	p= 0.000004		p=0.0006	p=0.0000227207	p=0.0000000628

C7				C13		
5 µM	10 µM	20 µM		5 µM	10 µM	20 µM
p=0.0000000000	p=0.0000000000	p=0.0000000000		p=0.0000481243	p=0.0000004392	p= 0.0000000141

IL-4_primary CD4+T cells_Dose_Response)
Statistics: Paired t-test

C7	
10 µM	20 µM
p=0.0346167987	p=0.0113768943

C13			
2.5 µM	5 µM	10 µM	20 µM
p=0.0297	p=0.0418	p=0.0106	p=0.00083188

Fig. 1F (IL-13_primary CD4+T cells_Dose_Response)
Statistics: Paired t-test

C7							
0 µM	0.25 µM	0.5 µM	1 µM	2.5 µM	5 µM	10 µM	20 µM
	p=0.021	p=0.006	p=0.007	p=0.002	p=0.004	p=0.001	p=0.0000150139

C13							
0 µM	0.25 µM	0.5 µM	1 µM	2.5 µM	5 µM	10 µM	20 µM
	p=0.015	p=0.004	p=0.006	p=0.006	p=0.00074	p=0.0000041	p=0.00000036

Fig. 3C (IL-13_Hut78_screening)
Statistics: Unpaired t-test

C7				C13		
5 µM	10 µM	20 µM		5 µM	10 µM	20 µM
p= 0.00003	p=0.000004	p=7.4E-09		p=0.00025	p=0.0001	p=5.8E-08

BP79				BP84		
5 µM	10 µM	20 µM		5 µM	10 µM	20 µM
p=3.2E-09	p=7.8E-08	p=2.5E-09		p=0.027	p=0.00241	p=0.0011

BP86				BP85		
5 µM	10 µM	20 µM		5 µM	10 µM	20 µM
p=0.026	p=0.00007	p=0.00009		p=0.004	p=0.0021	p=0.0009

BP87				BP95		
5 µM	10 µM	20 µM		5 µM	10 µM	20 µM
p=0.002	p=0.0003	p=0.001		p=0.03	p=0.0015	p=0.0009

BP96		
5 µM	10 µM	20 µM
p=0.0005	p=0.0002	p=0.0001

Fig. 4A (IL-4_Dose response_CD4+T cells)							
Statistics: Unpaired t-test							
	0.3 µM	0.6 µM	1.25 µM	2.5 µM	5 µM	10 µM	20 µM
	p=0.025	p=0.014	p=0.009	p=0.006	p=0.0002	p=0.0000000556	p=0.0000000005

Fig. 4B (IL-13_Dose response_CD4+T cells)							
Statistics: Unpaired t-test							
0.15 µM	0.3 µM	0.6 µM	1.25 µM	2.5 µM	5 µM	10 µM	20 µM
p=0.04	p=0.02	p=0.004	p=0.0003	p=0.00000	p=0.0000000107	p=0.0000000000	p=0.0000000000

Fig. 4F (TSLP mediated CD4+T cell proliferation)	
Statistics: Unpaired t-test	
TSLP_Day5	BP79_Day5
p=0.02	p=0.00006

Fig. 4G (Western blot_primary CD4+T cells_pSTAT6/STAT6)	
Statistics: One tailed, unpaired t-test	
TSLP	TSLP+BP79
p= 0.05	p= 0.04

Fig. 4H (Western blot_primary keratinocytes_pSTAT3/STAT3)	
Statistics: One tailed, paired t-test	
TSLP	TSLP+BP79
p= 0.016	p= 0.0018

Fig. 4J (CCL17 expression_primary dendritic cells)
Statistics: One tailed, unpaired t-test
TSLP+BP79
p=0.02

Fig. 4K (IL-13 % of expression in co-culture of primary dendritic cells and CD4+T cells)	
Statistics: Two tailed, unpaired t-test	
TSLP	TSLP+BP79
p= 0.05	p= 0.0009

Fig. 5A (PLA signal quantification)		
Statistics: Unpaired t-test		
	TSLP	TSLP+DMSO
TSLP+BP79	p= 0.0000[illegible]	p= 0.0000[illegible]

Fig. 6C (3D Immunocompetent skin tissue models: Cytokines expression)		
Statistics: Unpaired t-test		
Cytokine	Groups	
	FLG-/CD4+T cells	FLG-/CD4+T cells/BP79
TSLP	p=0.0000003164	p=0.0000000486
IL-4	p= 0.005	p=0.0000057083
IL-13	p=0.04	p= 0.0006870173
IL-5	p=0.00001	p=0.0000153542
IL-9	p=0.0000000026	p=0.0000000026
IL-22	p=0.0009	p=0.0000278718
IL-2	p=0.0000003853	p=0.0000003830
IFN gamma	p=0.0000000216	p= 0.0000000184
TNF alpha	p=0.51	p=0.0000000017

Fig. 7D (Atopic diseases-organ chip: Cytokines expression_ELISA)			
Statistics: Unpaired t-test			
Cytokines	Groups		
	Atopic condition_untreated	Atopic condition_BP79	Atopic condition_Tacrolimus
**IL-13**	p=0.0000005	p=0.0000003	p=0.0000062308
**IL-4**	p=0.0001	p=0.011	p=0.03
**TSLP**	p=0.019	p=0.06	p=0.016
**Periostin**	p=0.0006	p=0.00012	p=0.08

Fig. 7E (Atopic diseases-organ chip: mRNA expression_qPCR)			
Statistics: One tailed t-test			
Gene	Model	Groups	
		BP79	Tacrolimus
**TSLP**	**Skin**	p= 0.055	p=0.311
**FLG**	**Skin**	p= 0.05	p= 0.20
**KRT5**	**Skin**	p= 0.03	p= 1.9E-07
**KRT14**	**Skin**	p=0.045	p= 0.02
**KRT10**	**Skin**	p= 0.10	p= 0.04
**TSLP**	**Lung**	p= 0.03	p= 0.14

Fig. 8C (Cell count: Migrated CD4+T cells in the skin models)	
Statistics: One tailed t-test	
**BP79**	**Tacrolimus**
p= 0.046	p= 0.10

Appendix Fig. S1 (Optimization of TSLP mediated Th2 cytokines secretion assay [illegible]				
Statistics: Unpaired t-test				
	12 hours	24 hours	36 hours	48 hours
**IL-13**	p=0.82	p=0.05	p=0.009	p=0.01
**IL-4**	p=0.22	p= 0.87	p=0.047	p=0.11

2. Appendix Figures

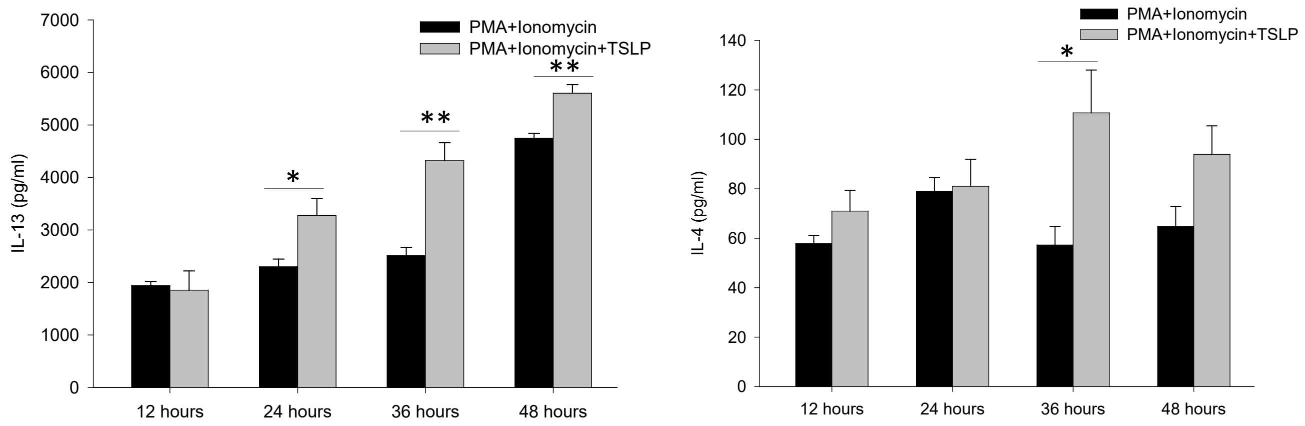

Appendix Figure S1. Optimization of TSLP mediated Th2 cytokines secretion assay condition. Bars represent the mean ± s.d., n=3. *p≤0.05, **p≤0.01, ***p≤0.001

A)

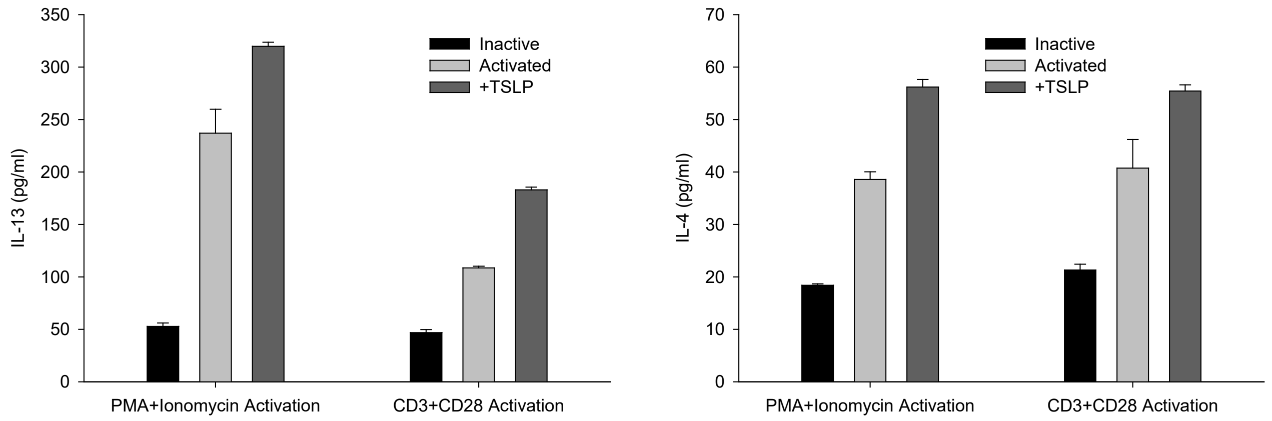

B)

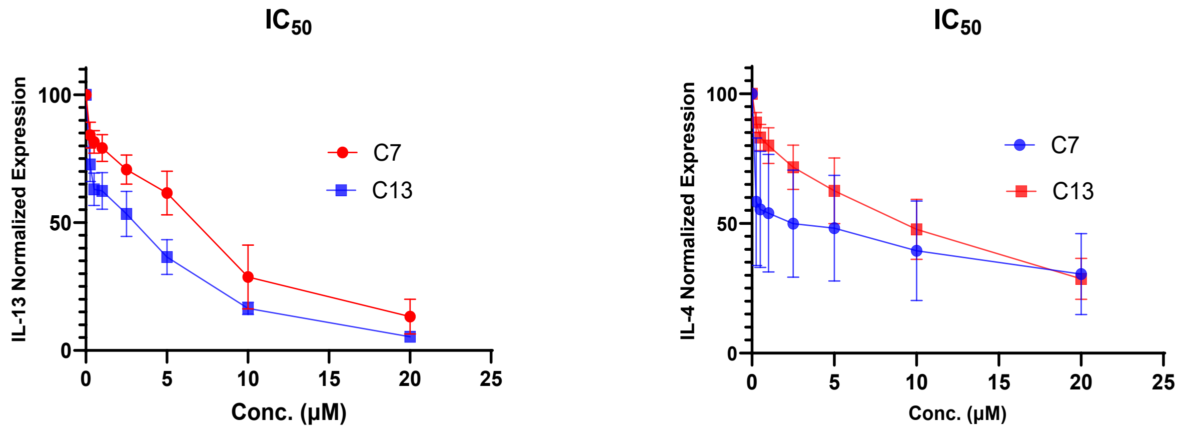

Appendix Figure S2. A) Optimization of TSLP mediated Th2 cytokines secretion assay condition. Bars represent the mean ± s.d., n=2. **B)** Dose-response curves of C7 and C13 which were used to determine the IC50 value and HillSlope.

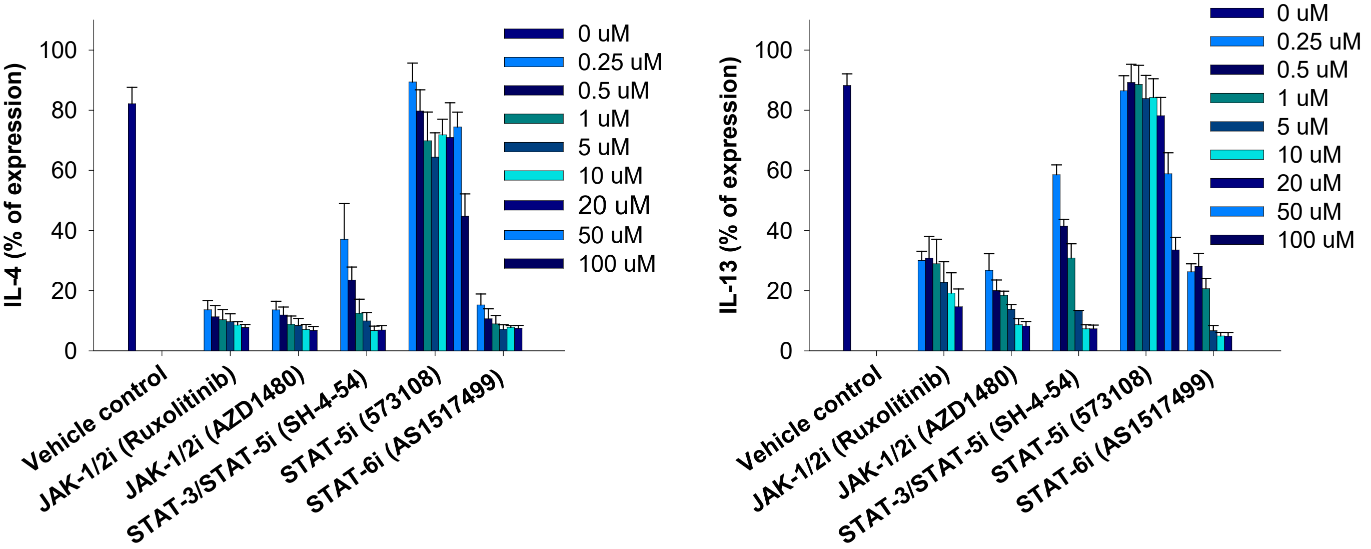

Appendix Figure S3. Mechanism of Th2 cytokine secretion. TSLP-activated primary CD4+T cells were treated with JAK1, JAK2, STAT3, STAT5 and STAT6 inhibitors. IL-4 and IL-13 secretion was detected with ELISA. Bars represent the mean ± s.d., n=3-5.

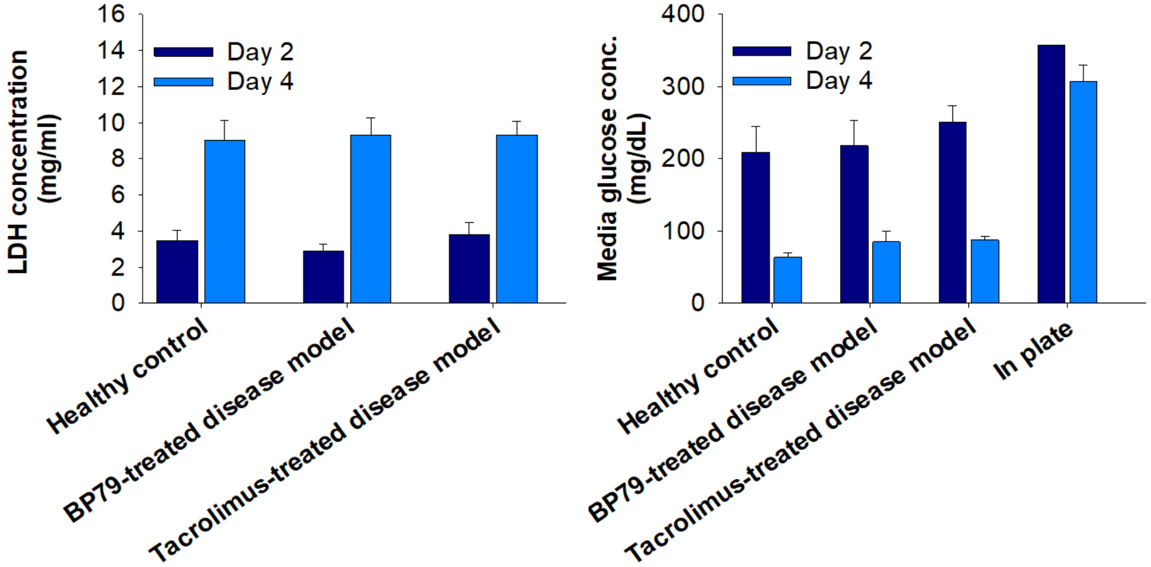

Appendix Figure S4. Except for LDH and glucose concentration fluctuations due to media changes, LDH and glucose levels in the OoC cultures indicate stable co-culture conditions.

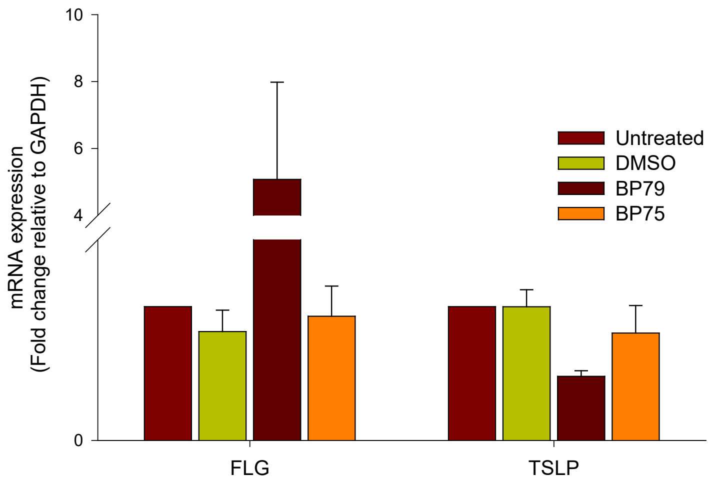

Appendix Figure S5. Primary keratinocytes from 5 donors were treated with the TSLP inhibitors or DMSO. Filaggrin and TSLP expression was determined using qPCR. BP79 induced filaggrin and reduced TSLP expression.

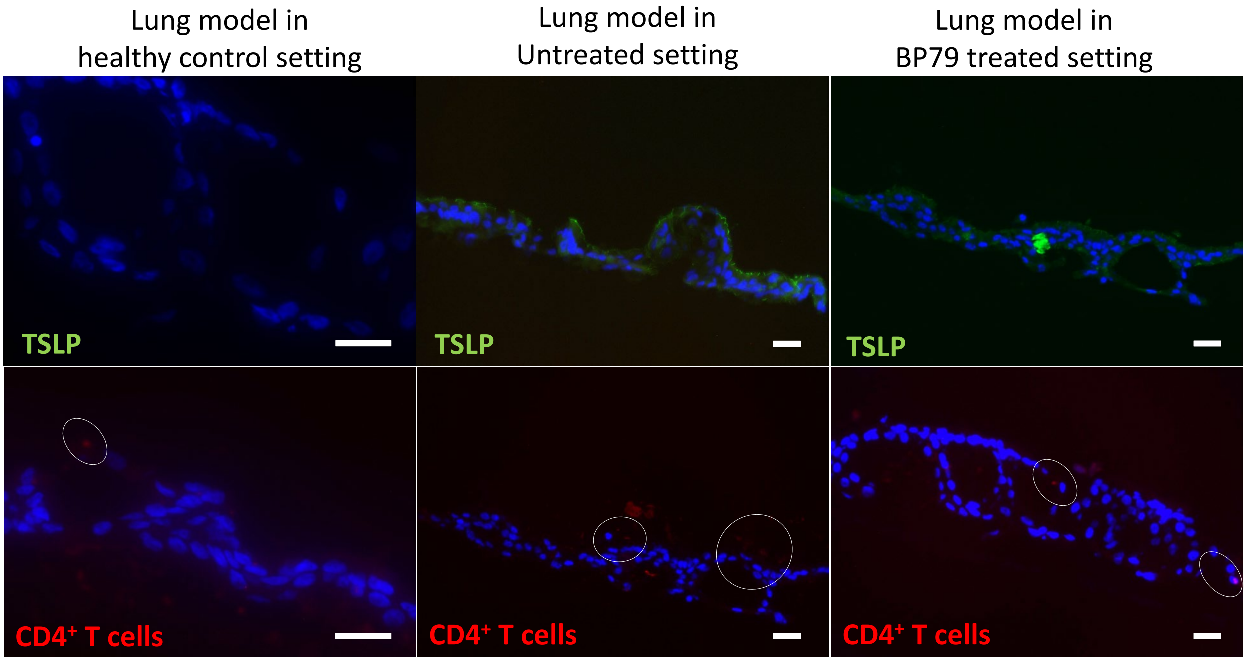

Appendix Figure S6. Compound efficacy testing using Atopic Dermatitis-on-a-chip model. Healthy and atopic dermatitis skin tissue model and healthy bronchial epithelial model were mounted on a chip. BP79 was applied topically on the skin model. BP79 treatment reduced TSLP expression in the bronchial epithelial model compared to that of the untreated group. Slight CD4+T cell migration was observed in both groups.

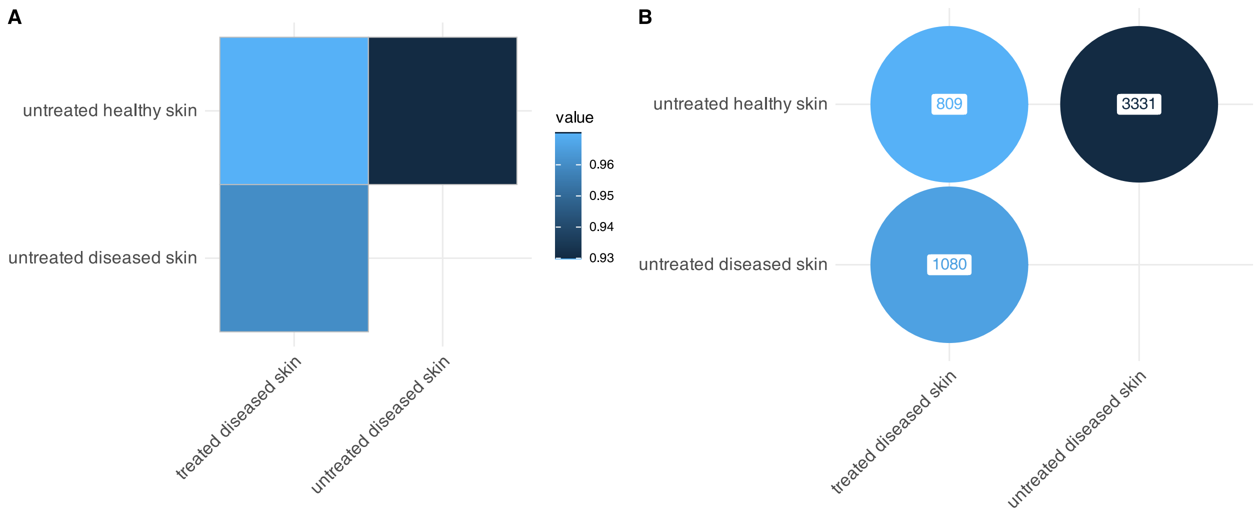

Appendix Figure S7. Treated diseased skin is more similar to untreated healthy skin than to treated healthy skin: **A**, Spearman correlation between averaged expression values for the three groups: healthy skin, untreated; diseased skin, untreated; diseased skin, treated. **B**, number of DEGs at FDR < 0.01 and absolute log2 FC > 1 (at least two-fold change).

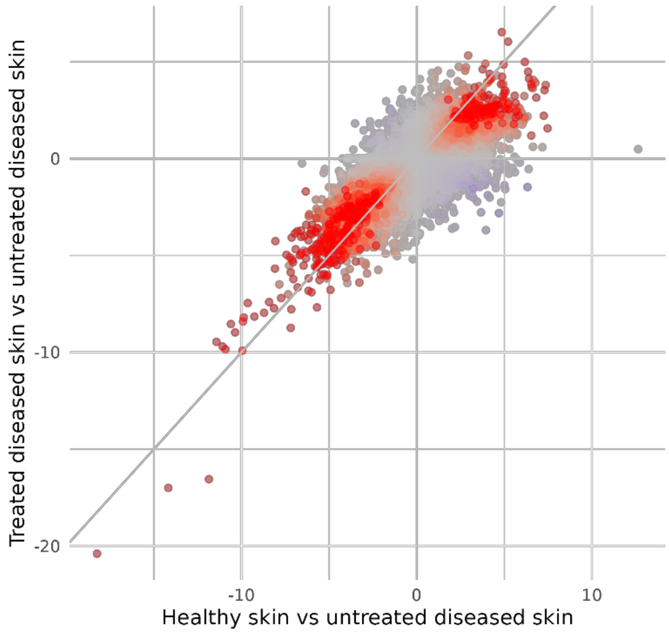

Appendix Figure S8. Log2 fold change of genes in untreated diseased skin vs healthy skin (x-axis) and untreated diseased skin vs treated diseased skin (y-axis). Higher log2 fold change values signify higher expression in the first condition of the comparison. Red corresponds to genes showing change in the same direction in both comparisons, blue to genes showing change in the opposite direction. Color intensity corresponds to the degree of agreement (discordance score, Domaszewska et al. (2017)). The Pearson correlation coefficient between the two comparisons is 0.74 and significant (p indistinguishable from 0).

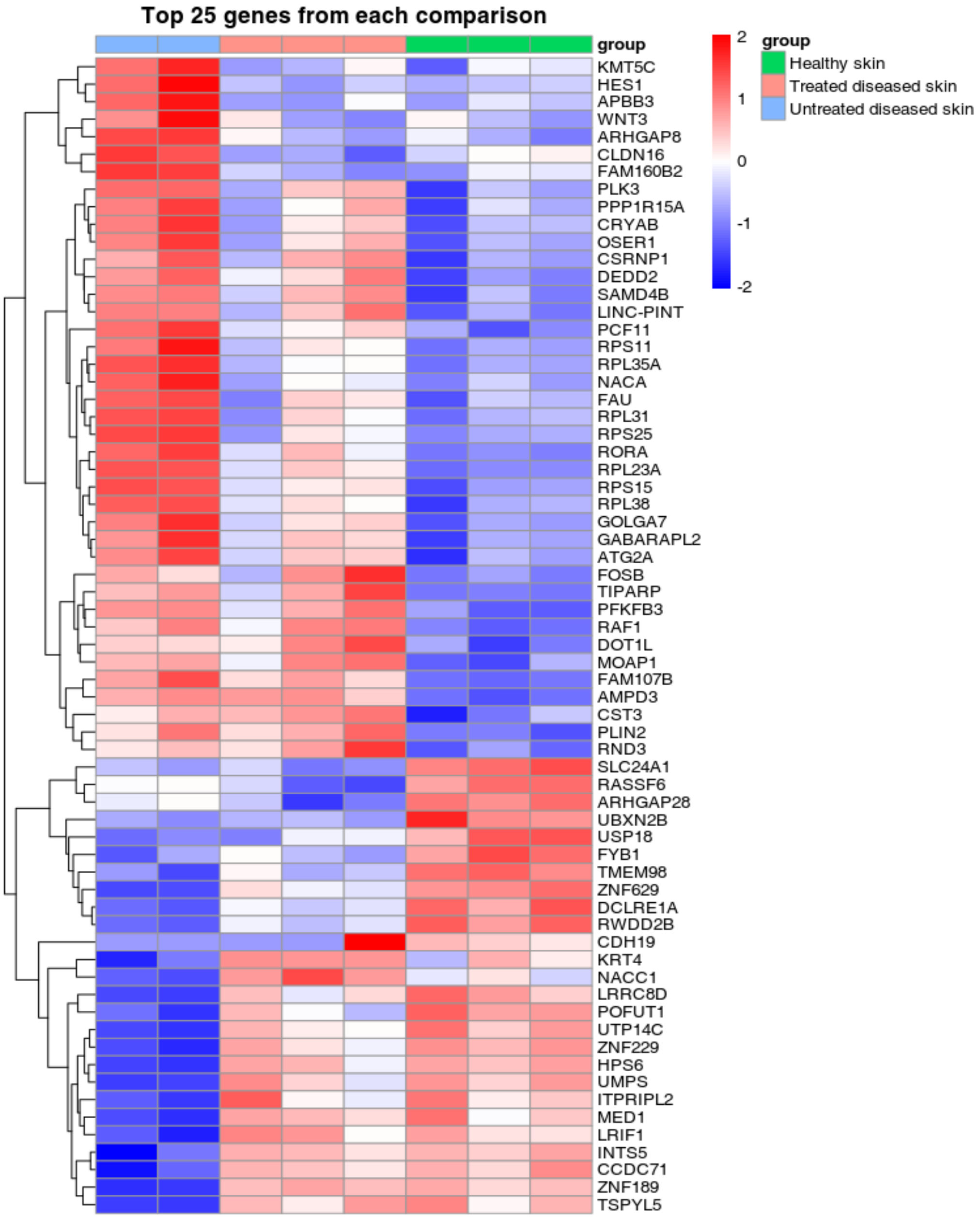

Appendix Figure S9. Heatmap showing gene expression of top 25 genes from each comparison.

3. Appendix Synthetic Protocols and Characterization:

General Protocol A: Synthesis of 2-chloro-N-phenyl acetamides

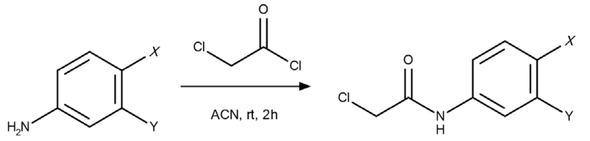

Chloroacetyl chloride (2 equiv.) was added dropwise to a stirred solution of 3,4-dichloroaniline (or tert-butyl-4-aminobenzoate, 1 equiv.) dissolved in acetonitrile. The reaction was stirred for 2 h and concentrated under reduced pressure. The mixture was diluted with water and extracted with ethyl acetate. 2-chloro-N-(3,4-dichlorophenyl)acetamide (or tert-butyl 4-(2-chloroacetamido)benzoate) was used without further purification.

General Protocol B: Synthesis of substituted N-phenyl acetamides

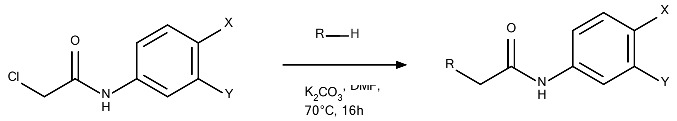

The nucleophile (2 equiv.) was stirred with K_2CO_3 (3 equiv.) in anhydrous DMF for 5 mins. (1 equiv.) -chloro-N-(3,4-dichlorophenyl)acetamide (or tert-butyl 4-(2-chloroacetamido)benzoate) was added, and the mixture was stirred at 70 °C overnight. The reaction mixture was diluted with water water and extracted with ethyl acetate. The product was purified by flash chromatography using a mixture of hexane and ethyl acetate.

General Protocol C: Amide coupling.

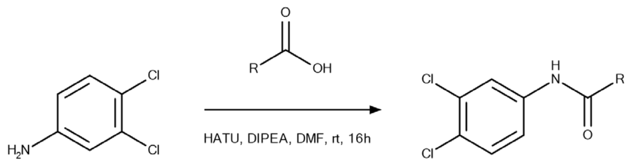

The carboxylic acid (1 equiv.) was dissolved in DMF and activated with HATU (2 equiv.) in the presence of DIPEA (2 equiv.) for 15 mins. 3,4-Dichloroaniline (2 equiv.) was subsequently added and the reaction was stirred for 16h at room temperature. The reaction mixture was diluted with water and the product was extracted by ethyl acetate. The product was purified by flash chromatography using a mixture of hexane and ethyl acetate.

General Protocol D: Ring-opening condensation

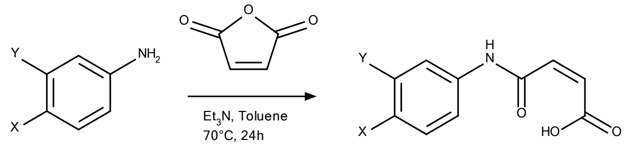

3,4-dichloroaniline (or 4-phenoxyaniline, 1 equiv.) and maleic anhydride were dissolved in toluene. Triethylamine (1.1 equiv.) was added to the mixture and stirred for 24h at 70 ˚C. The product was purified by flash chromatography using a mixture of DCM and methanol.

General Protocol E: Synthesis of BP79 Analogues.

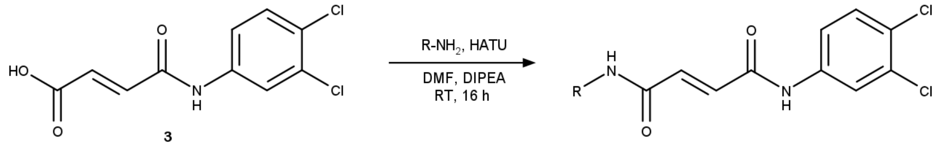

Intermediate 3 (1 equiv.) was placed in a round-bottom flask and sealed with a rubber septum. The flask was vented with N_2. Anhydrous DMF was injected into the flask and the solution was stirred. DIPEA (2 equiv.) was injected into the flask and stirred. The amine (1 equiv.) was dissolved in anhydrous DMF and injected into the flask. HATU (1.1 equiv.) was dissolved in warmed anhydrous DMF and injected into the flask. The reaction was stirred for 16h at room temperature. The reaction was poured into water and extracted by ethyl acetate. The organic layer was washed with saturated sodium bicarbonate, water, and brine. The organic layer was dried by $MgSO_4$ and gravity filtered. The product was purified by automated flash chromatography using a mixture of hexane and ethyl acetate.

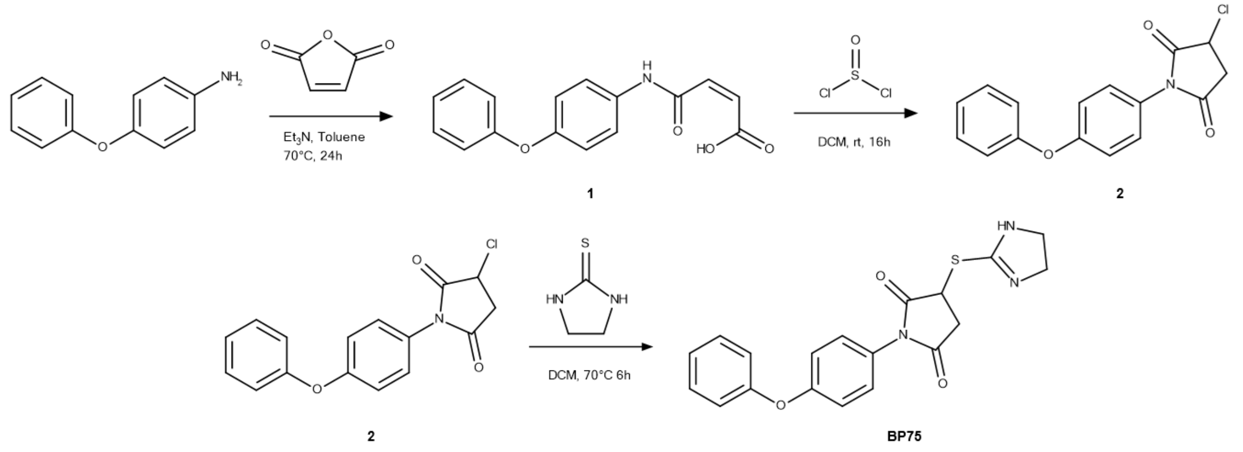

Scheme 1: Synthesis of **BP75**.

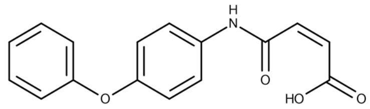

Intermediate 1. (Z)-4-oxo-4-((4-phenoxyphenyl)amino)but-2-enoic acid was prepared as outlined in general procedure D on a 2.807mmol scale.

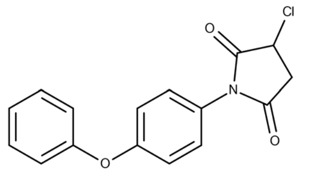

Intermediate 2. (Z)-4-oxo-4-((4-phenoxyphenyl)amino)but-2-enoic acid (4.014mmol, 1 equiv.) was dissolved in DCM. Thionyl chloride (80.280mmol, 20 equiv.) was added dropwise to the solution and the reaction was stirred for 16h at room temperature. 3-chloro-1-(4-phenoxyphenyl)pyrrolidine-2,5-dione was purified by flash chromatography using a mixture of hexane and ethyl acetate. ^{1}H NMR (400 MHz, $CDCl_3$) δ 7.47 – 7.34 (m, 3H), 7.17 (t, *J* = 7.6 Hz, 1H), 7.12 – 6.95 (m, 5H), 4.73 (dd, *J* = 8.7, 4.1 Hz, 1H), 3.40 (dd, *J* = 18.9, 8.7 Hz, 1H), 3.02 (dd, *J* = 18.9, 4.1 Hz, 1H). ^{13}C NMR (101 MHz, $CDCl_3$) δ 171.93, 158.16, 156.21, 132.41, 130.36, 130.03, 124.12, 120.77, 119.54, 118.95, 116.51, 49.03, 39.33. (752.3 mg, 62% yield).

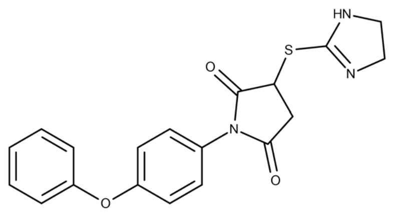

BP75. 3-chloro-1-(4-phenoxyphenyl)pyrrolidine-2,5-dione (2.025mmol, 1 equiv.) and 2-imidazolidinethione (4.050mmol, 2 equiv.) were dissolved in DMF and stirred for 6h at 70 ˚C. The reaction was diluted with water and the product was extracted by ethyl acetate. 3-((4,5-dihydro-1H-imidazol-2-yl)thio)-1-(4-phenoxyphenyl)pyrrolidine-2,5-dione was purified by flash chromatography using a mixture of hexane and ethyl acetate. ^{1}H NMR (400 MHz, DMSO) δ 10.26 (s, 1H), 7.45 – 7.36 (m, 2H), 7.35 – 7.26 (m, 3H), 7.16 (t, *J* = 7.4 Hz, 1H), 7.05 – 6.98 (m, 2H), 6.75 – 6.68 (m, 1H), 4.88 (dd, *J* = 10.0, 3.5 Hz, 1H), 4.17 (td, *J* = 8.2, 1.9 Hz, 2H), 3.63 (td, *J* = 8.5, 2.3 Hz, 2H), 3.24 (dd, *J* = 16.9, 3.6 Hz, 1H), 3.02 (dd, *J* = 17.0, 10.0 Hz, 1H). ^{13}C NMR (101 MHz, DMSO) δ 168.59, 167.30, 160.94, 157.54, 156.86, 140.63, 130.67, 130.55, 124.10, 119.31, 114.44, 113.97, 109.51, 61.18, 51.85, 41.77. HPLC-MS t_R = 7.84 minutes, 91.2% purity, [$C_{19}H_{17}N_3O_3S$ + H] = 369.1, found 368.7 (325.8mg, 40% yield).

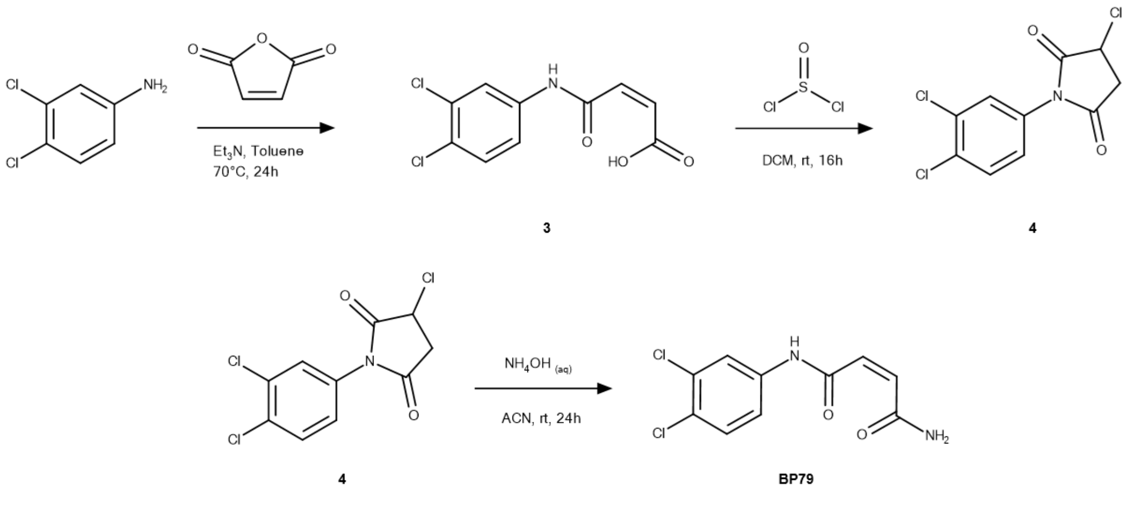

Scheme 2: Synthesis of **BP79**.

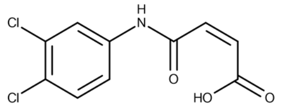

Intermediate 3. (Z)-4-((3,4-dichlorophenyl)amino)-4-oxobut-2-enoic acid was prepared as outlined in general procedure 4 on a 1.020mmol scale (221.0mg, 90% yield). ^{1}H NMR (400 MHz, Acetone) δ 7.98 (d, *J* = 2.3 Hz, 1H), 7.42 (dd, *J* = 8.8, 2.4 Hz, 1H), 7.35 (d, *J* = 8.7 Hz, 1H), 6.20 (d, *J* = 13.2 Hz, 1H), 6.07 – 5.98 (m, 1H).

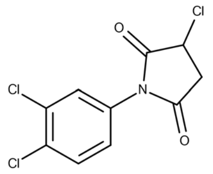

Intermediate 4. (Z)-4-((3,4-dichlorophenyl)amino)-4-oxobut-2-enoic acid (6.031mmol, 1 equiv.) was dissolved in DCM. Thionyl chloride (206.522mmol, 34 equiv.) was added dropwise to the solution and the reaction was stirred for 16h at room temperature. 3-chloro-1-(3,4-dichlorophenyl)pyrrolidine-2,5-dione was purified by flash chromatography using a mixture of hexanes and ethyl acetate. ^{1}H NMR (400 MHz, $CDCl_3$) δ 7.49 (d, *J* = 1.2 Hz, 1H), 7.49 – 7.39 (m, 1H), 7.23 – 7.11 (m, 1H), 4.72 (dd, *J* = 8.8, 1.3 Hz, 1H), 3.42 (dd, *J* = 19.0, 1.3 Hz, 1H), 3.02 (dd, *J* = 19.0, 1.2 Hz, 1H). (1546.5mg, 92% yield).

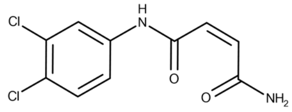

BP79. 3-chloro-1-(3,4-dichlorophenyl)pyrrolidine-2,5-dione (1.175mmol, 1 equiv.) was added to a solution of 3mL ACN and 1.5mL ammonium hydroxide (30-33%) and stirred for 24h at room temperature. The crude precipitate was isolated by vacuum filtration. N^1-(3,4-dichlorophenyl)maleamide was purified by flash chromatography using a mixture of hexane and ethyl acetate. ^{1}H NMR (400 MHz, DMSO) δ 11.30 (s, 1H), 8.01 (d, *J* = 2.4 Hz, 1H), 7.95 (s, 1H), 7.57 (d, *J* = 8.7 Hz, 1H), 7.49 – 7.42 (m, 2H), 6.28 (s, 2H). ^{13}C NMR (101 MHz, DMSO) δ 167.08, 164.38, 139.44, 132.69, 132.04, 131.50, 131.19, 125.33, 120.90, 119.77.

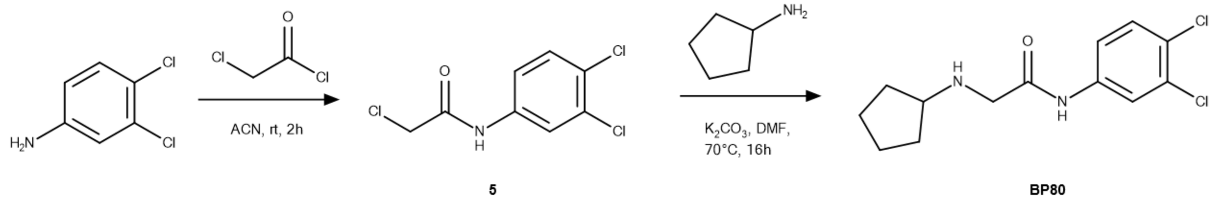

Scheme 3: Synthesis of **BP80**

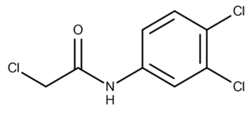

Intermediate 5. 2-chloro-N-(3,4-dichlorophenyl)acetamide was prepared as outlined in general procedure A on a 5.987mmol scale (1427.8mg, 97% yield). ^{1}H NMR (400 MHz, Acetone) δ 9.52 (s, 1H), 7.92 (d, *J* = 2.4 Hz, 1H), 7.48 – 7.36 (m, 2H), 4.13 (s, 2H).

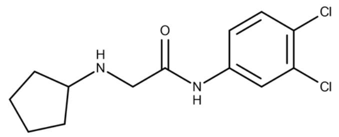

BP80. 2-(cyclopentylamino)-N-(3,4-dichlorophenyl)acetamide was prepared as outlined in general procedure B using cyclopentylamine on a 0.419mmol scale. ^{1}H NMR (400 MHz, $CDCl_3$) δ 9.51 (s, 1H), 7.83 (d, *J* = 2.4 Hz, 1H), 7.43 (dd, *J* = 8.7, 2.4 Hz, 1H), 7.38 (d, *J* = 8.7 Hz, 1H), 3.38 (s, 2H), 3.15 (p, *J* = 6.2 Hz, 1H), 1.92 – 1.79 (m, 2H), 1.79 – 1.67 (m, 2H), 1.62 (dq, *J* = 8.8, 5.8 Hz, 2H), 1.45 – 1.32 (m, 2H). ^{13}C NMR (101 MHz, $CDCl_3$) δ 170.63, 137.21, 132.75, 130.50, 127.08, 120.90, 118.54, 60.66, 51.67, 32.99, 23.69. HPLC-MS t_R = 7.08 minutes, 97.5% purity, m/z calculated for [$C_{13}H_{16}Cl_2N_2O$ + H] = 287.07, found 287.0 (93.5mg, 78% yield).

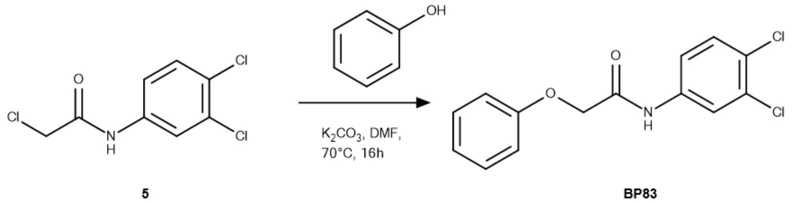

Scheme 4: Synthesis of **BP83**

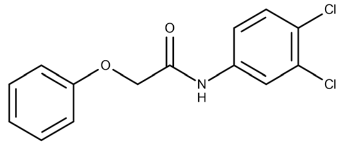

BP83. N-(3,4-dichlorophenyl)-2-phenoxyacetamide was prepared as outlined in general procedure B using phenol and **intermediate 5** on a 0.419mmol scale. ^{1}H NMR (400 MHz, $CDCl_3$) δ 8.36 (s, 1H), 7.86 (d, *J* = 2.4 Hz, 1H), 7.48 – 7.34 (m, 4H), 7.10 (t, 1H), 7.00 (d, *J* = 1.2 Hz, 2H), 4.63 (s, 2H). ^{13}C NMR (101 MHz, $CDCl_3$) δ 166.43, 156.82, 136.31, 132.93, 130.62, 130.00, 128.15, 122.70, 121.76, 119.30, 114.85, 67.55. HPLC-MS t_R = 9.54 minutes, 99.6% purity, [$C_{14}H_{11}Cl_2NO_2$ + H] = 296.02, found 296.0 (94.5mg, 76% yield).

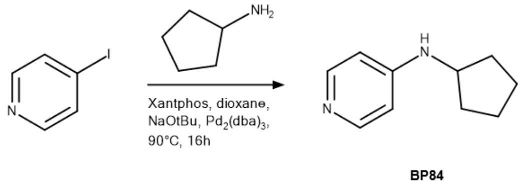

Scheme 5: Synthesis of **BP84**.

BP84. Iodopyridine (0.488mmol, 1 equiv.), sodium tert-butoxide (1.951mmol, 4 equiv.), and 9,9-dimethyl-4,5-bis(diphenylphosphino) xanthene (Xantphos, 0.024mmol, 0.05 equiv.) were placed in a nitrogen-flushed RBF and suspended in dioxane (5 mL) which was previously degassed by bubbling with nitrogen. This suspension was degassed by bubbling with nitrogen. Tris(dibenzylideneacetone)dipalladium (0) (Pd2[dba]$_3$, 0.015mmol, 0.03 equiv.) was added, after which the suspension was again degassed by bubbling with nitrogen. Cyclopentylamine (0.488mmol, 1 equiv.) was added and the suspension was degassed by bubbling with nitrogen for 10 minutes. The tube was sealed, and the reaction was stirred at 90 °C overnight. The resulting suspension was filtered through celite and washed with methanol. N-cyclopentylpyridin-4-amine was purified by reverse phase chromatography using a mixture of methanol and water. ^{1}H NMR (400 MHz, MeOD) δ 7.96 (d, *J* = 6.4 Hz, 2H), 6.53 (d, *J* = 6.7 Hz, 2H), 3.82 (p, *J* = 5.3 Hz, 1H), 2.08 – 1.95 (m, 2H), 1.81 – 1.70 (m, 2H), 1.69 – 1.59 (m, 2H), 1.58 – 1.48 (m, *J* = 5.1 Hz, 2H). ^{13}C NMR (101 MHz, MeOD) δ 154.43, 147.85, 107.40, 53.21, 32.28, 23.53. HPLC-MS t_R = 3.87 minutes, 95.2% purity [$C_{10}H_{14}N_2$ + H] = 163.1, found 163.1 (58.4mg, 74% yield).

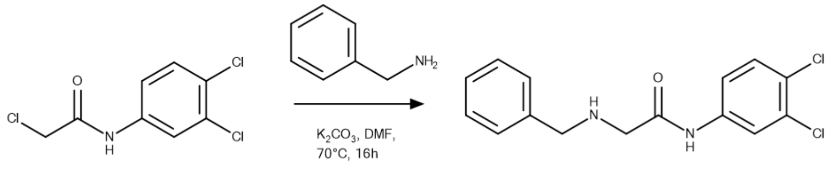

5 BP85

Scheme 6: Synthesis of **BP85**

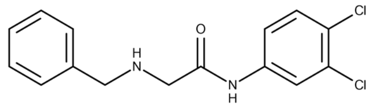

BP85. 2-(benzylamino)-N-(3,4-dichlorophenyl)acetamide was prepared as outlined in general procedure B using benzylamine and **intermediate 5** on a 0.419mmol scale. ^{1}H NMR (400 MHz, $CDCl_3$) δ 9.36 (s, 1H), 7.79 (d, *J* = 2.0 Hz, 1H), 7.44 – 7.25 (m, 8H), 3.86 (s, 2H), 3.44 (s, 2H). ^{13}C NMR (101 MHz, $CDCl_3$) δ 169.81, 138.86, 137.07, 132.76, 130.50, 128.84, 128.12, 127.82, 127.71, 127.45, 127.24, 121.04, 118.63, 77.24, 54.18, 52.38, 42.23. HPLC-MS t_R = 7.40 minutes, 96.5% purity, [$C_{15}H_{14}Cl_2N_2O$ + H] = 309.05, found 309.0 (111.7mg, 86% yield).

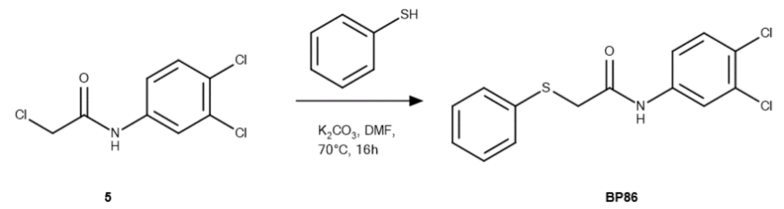

Scheme 7: Synthesis of **BP86**.

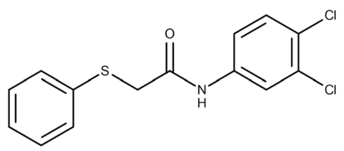

BP86. N-(3,4-dichlorophenyl)-2-(phenylthio)acetamide was prepared as outlined in general procedure B using thiophenol and **intermediate 5** on a 0.419mmol scale. ^{1}H NMR (400 MHz, $CDCl_3$) δ 8.62 (s, 1H), 7.74 (d, *J* = 2.4 Hz, 1H), 7.39 – 7.27 (m, 6H), 3.78 (s, 2H). ^{13}C NMR (101 MHz, $CDCl_3$) δ 166.23, 136.69, 133.73, 132.89, 130.55, 129.62, 128.54, 128.04, 127.34, 121.52, 119.04, 38.47. HPLC-MS t_R = 9.56 minutes, 90.9% purity, [$C_{15}H_{14}Cl_2NOS$ - H] = 310.0, found 309.0 (80.6mg, 62% yield)

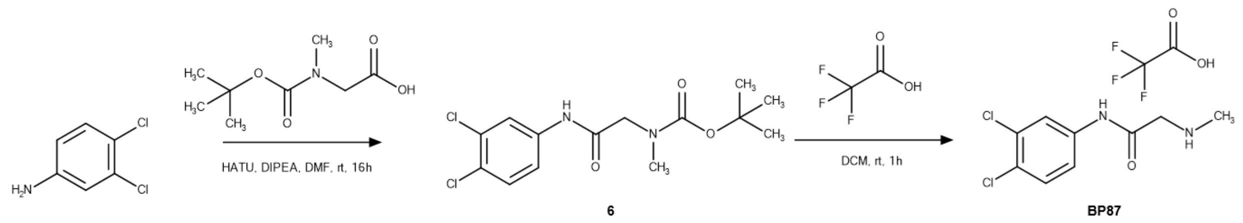

Scheme 8: Synthesis of **BP87**

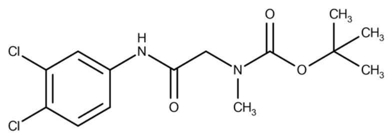

Intermediate 6. tert-butyl (2-((3,4-dichlorophenyl)amino)-2-oxoethyl)(methyl)carbamate was prepared as outlined in general procedure C using N-(tert-butoxycarbonyl)-N-methylglycine on a 1.057mmol scale (296.3mg, 84% yield).

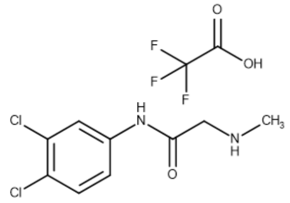

BP87. *N*-(3,4-dichlorophenyl)-2-(methylamino)acetamide (0.535mmol) was dissolved in a 1:1 ratio of DCM:TFA solution and stirred for 1h. The reaction mixture was concentrated under reduced pressure to afford N-(3,4-dichlorophenyl)-2-(methylamino)acetamide trifluoroacetic acid salt. ^{1}H NMR (400 MHz, DMSO) δ 10.89 (s, 1H), 8.95 (s, 2H), 7.95 (s, 1H), 7.63 (d, *J* = 8.8 Hz, 1H), 7.51 (d, *J* = 8.8 Hz, 1H), 3.97 (s, 2H), 2.65 (s, 3H). ^{13}C NMR (101 MHz, DMSO) δ 165.03, 159.06, 158.74, 138.60, 131.67, 131.44, 126.03, 121.04, 119.88, 50.16, 40.13, 39.91, 33.22. HPLC-MS t_R = 7.08 minutes, 99.6% purity, [$C_9H_{10}Cl_2N_2O$ - H] = 231.0, found 231.0 (130.2mg, 70% yield).

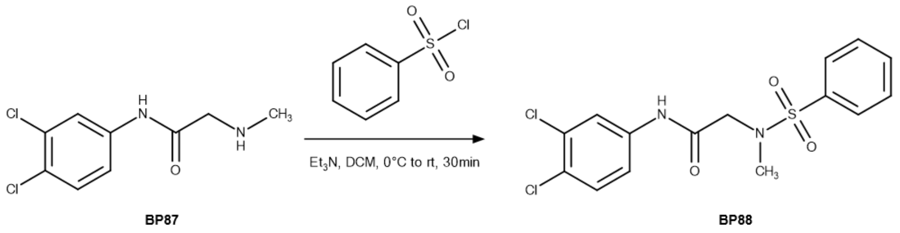

Scheme 9. Synthesis of **BP88**

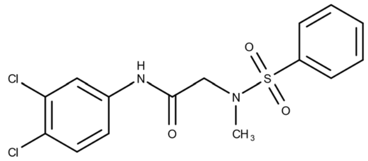

BP88. N-(3,4-dichlorophenyl)-2-(methylamino)acetamide (**BP87**) (0.288mmol, 1 equiv.) was dissolved in DCM. Triethylamine (0.576mmol, 2 equiv.) was added to the mixture and the reaction was cooled to 0 °C. Benzenesulfonyl chloride (0.576mmol, 2 equiv.) was added dropwise and stirred from 0 °C to room temperature for 30 mins. The product was purified by column chromatography using a hexane and ethyl acetate mixture. ^{1}H NMR (400 MHz, $CDCl_3$) δ 8.47 (s, 1H), 7.89 – 7.82 (m, 3H), 7.76 – 7.66 (m, 1H), 7.66 – 7.58 (m, 2H), 7.42 (t, *J* = 1.3 Hz, 2H), 3.75 (s, 2H), 2.91 (s, 2H). ^{13}C NMR (101 MHz, $CDCl_3$) δ 165.94, 136.48, 135.63, 133.75, 132.92, 130.58, 129.61, 128.17, 127.63, 121.76, 119.26, 54.95, 37.40. HPLC-MS t_R = 9.27 minutes, 95.8% purity, [$C_{15}H_{14}N_2O_3S$ + H] = 373.0, found 372.9 (54.8mg, 51% yield).

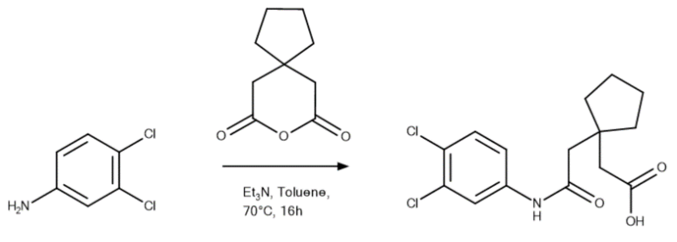

BP89

Scheme 10: Synthesis of **BP89**.

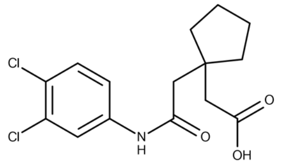

BP89. 2-(1-(2-((3,4-dichlorophenyl)amino)-2-oxoethyl)cyclopentyl)acetic acid was prepared as outlined in general procedure D on a 1.234mmol scale using 3,3-tetramethyleneglutaric anhydride (1.2 equiv.) and purifying by flash chromatography using a mixture of hexane, ethyl acetate, DCM, and methanol. ^{1}H NMR (400 MHz, MeOD) δ 7.91 (s, 1H), 7.47 – 7.40 (m, 2H), 2.59 (s, 2H), 2.55 (s, 2H), 1.77 – 1.67 (m, 8H). ^{13}C NMR (101 MHz, MeOD) δ 175.03, 171.71, 138.39, 131.89, 130.10, 126.25, 121.13, 119.15, 44.24, 43.58, 41.98, 37.63, 23.51. HPLC-MS t_R = 9.14 minutes, 100% purity, [$C_{15}H_{17}Cl_2NO_3$ + H] = 330.1, found 329.9 (211.8mg, 55% yield).

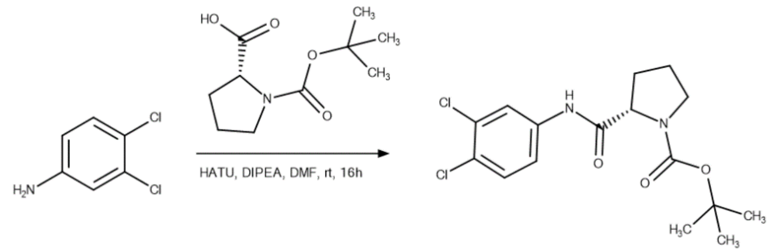

BP90

Scheme 11: Synthesis of **BP90**.

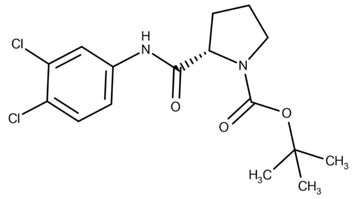

BP90. tert-butyl (S)-2-((3,4-dichlorophenyl)carbamoyl)pyrrolidine-1-carboxylate was prepared as outlined in general procedure C on a 1.394mmol scale using 1.5 equivalents of HATU, DIPEA, and 3,4-dichlroaniline. ^{1}H NMR (400 MHz, $CDCl_3$) δ 9.81 (s, 1H), 7.77 (s, 1H), 7.23 (s, 2H), 4.49 (s, 1H), 3.52 (t, *J* = 8.4 Hz, 1H), 3.41 (s, 1H), 2.39 (s, 1H), 2.10 – 1.88 (m, 3H), 1.52 (s, 9H). ^{13}C NMR (101 MHz, $CDCl_3$) δ 170.49, 137.98, 132.51, 130.17, 121.09, 118.63, 81.10, 77.24, 60.51, 47.31, 28.42, 27.82, 24.56. HPLC-MS t_R = 9.38 minutes, 99.6% purity, [$C_{16}H_{20}Cl_2N_2O_3$ + H] = 359.1, found 259.0 (489.0mg, 97% yield).

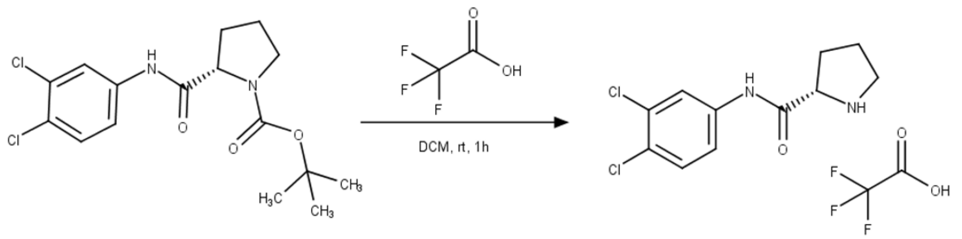

BP90 **BP91**

Scheme 12: Synthesis of **BP91**.

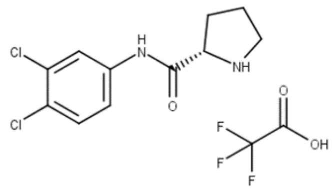

BP91. tert-butyl (S)-2-((3,4-dichlorophenyl)carbamoyl)pyrrolidine-1-carboxylate (**BP90**), 1.118mmol, 1 equiv.) was dissolved in a 1:1 ratio of DCM:TFA solution (10mL) and stirred for 1h. The reaction mixture was concentrated under reduced pressure to afford (S)-N-(3,4-dichlorophenyl)pyrrolidine-2-carboxamide trifluoroacetic acid salt. ^{1}H NMR (400 MHz, DMSO) δ 11.12 (s, 1H), 10.00 (s, 1H), 8.77 (s, 1H), 8.31 (s, 1H), 7.95 (s, 1H), 7.52 (s, 2H), 4.44 (t, *J* = 7.2 Hz, 1H), 3.32 (dd, *J* = 12.8, 6.1 Hz, 2H), 2.41 (dt, *J* = 13.9, 6.8 Hz, 1H), 2.08 – 1.85 (m, 3H). ^{13}C NMR (101 MHz, $CDCl_3$) δ 166.97, 136.37, 132.83, 130.56, 128.60, 121.61, 119.12, 60.33, 47.09, 30.21, 24.73. HPLC-MS t_R = 6.79 minutes, 96.7% purity, [$C_{11}H_{12}Cl_2N_2O$ - H] = 257.0, found 257.0 (> 100% yield).

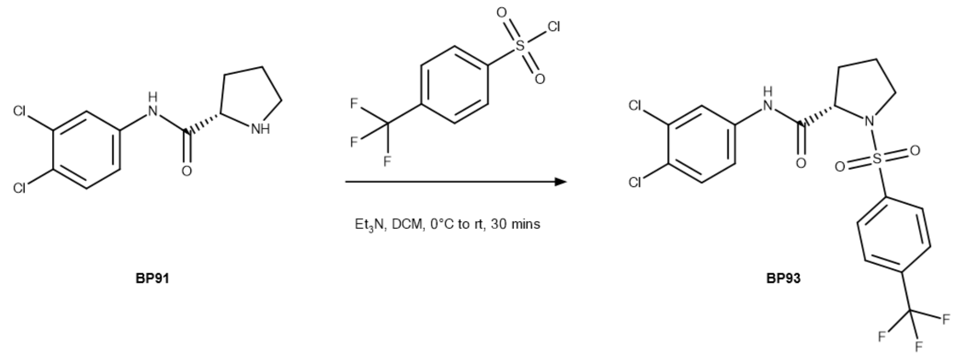

Scheme 13: Synthesis of **BP93**.

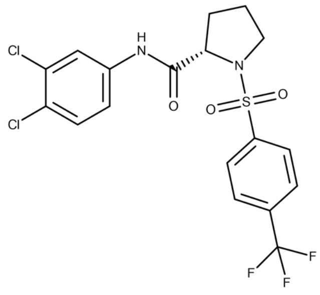

BP93. (S)-N-(3,4-dichlorophenyl)pyrrolidine-2-carboxamide trifluoroacetic acid salt (**BP91**, 0.268mmol, 1 equiv.) was dissolved in DCM with triethylamine (0.536mmol, 2 equiv.) and cooled to 0 ˚C. 4-Trifluoromethyl)benzenesulfonyl chloride (0.536mmol, 2 equiv.) was added and stirred from 0 ˚C to room temperature for 30 mins. (S)-N-(3,4-dichlorophenyl)-1-((4-(trifluoromethyl)phenyl)sulfonyl)pyrrolidine-2-carboxamide was purified by column chromatography using a mixture of hexane and ethyl acetate. ^{1}H NMR (400 MHz, $CDCl_3$) δ 8.66 (s, 1H), 7.95 (d, *J* = 8.1 Hz, 2H), 7.83 – 7.75 (m, 3H), 7.33 (s, 2H), 4.11 (dd, *J* = 8.7, 2.7 Hz, 1H), 3.61 (ddd, *J* = 10.4, 7.2, 3.3 Hz, 1H), 3.16 (td, *J* = 9.8, 6.8 Hz, 1H), 2.36 – 2.25 (m, 1H), 1.88 – 1.49 (m, 3H). ^{13}C NMR (101 MHz, $CDCl_3$) δ 168.64, 138.99, 136.76, 135.64, 135.31, 132.88, 130.54, 128.44, 128.00, 126.72, 121.69, 119.23, 62.97, 50.25, 29.63, 24.48. HPLC-MS t_R = 9.95 minutes, 99.6% purity, [$C_{18}H_{15}Cl_2F_3N_2O_3S$ + H] = 467.0, found 466.9 (87.7mg, 70% yield).

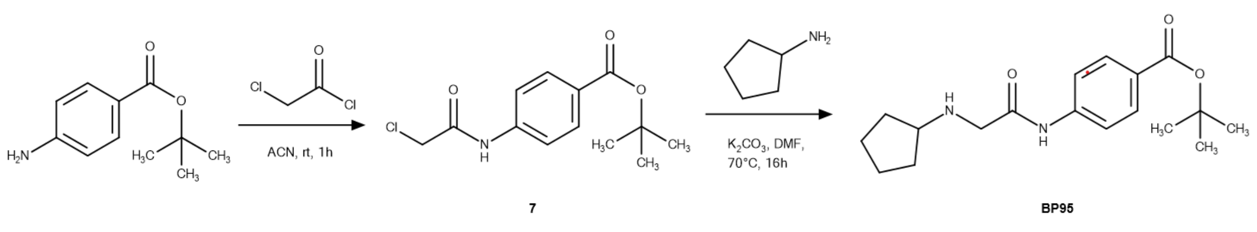

Scheme 14: Synthesis of **BP95**.

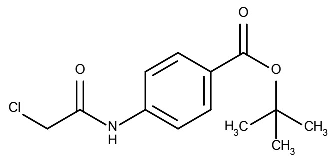

Intermediate 7. tert-butyl 4-(2-chloroacetamido)benzoate was prepared as outlined in general procedure A on a 5.175mmol scale but using 1.5mol equivalent of 2-chloroacetyl chloride, run for 1 hour, and purified by flash chromatography using a mixture of hexane and ethyl acetate (543mg, 39% yield).

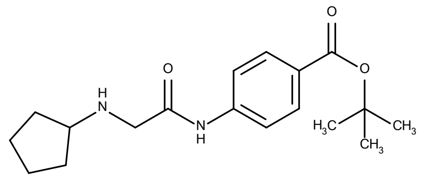

BP95. tert-butyl 4-(2-(cyclopentylamino)acetamido)benzoate was prepared as outlined in general procedure B using cyclopentylamine on a 0.371mmol scale. ^{1}H NMR (400 MHz, MeOD) δ 7.93 (d, 2H), 7.70 (d, *J* = 8.7 Hz, 2H), 3.42 (s, 2H), 3.13 (p, *J* = 6.6 Hz, 1H), 1.93 – 1.84 (m, 2H), 1.78 – 1.72 (m, 2H), 1.60 (s, 11H), 1.49 – 1.36 (m, 2H). ^{13}C NMR (101 MHz, MeOD) δ 171.18, 165.55, 142.19, 130.01, 126.94, 118.51, 80.68, 59.54, 50.85, 32.12, 27.06, 23.48. HPLC-MS t_R = 7.40 minutes, 92.9% purity. [$C_{18}H_{26}N_2O_3$ - H] = 317.2, found 317.1 (108.1mg, 92% yield).

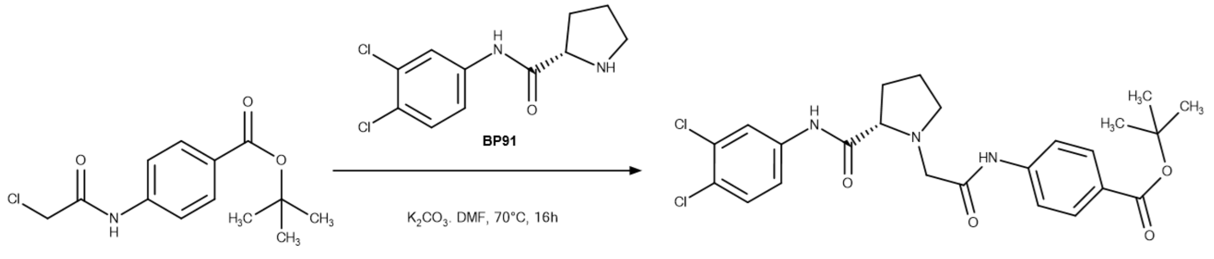

Scheme 15: Synthesis of **BP96**

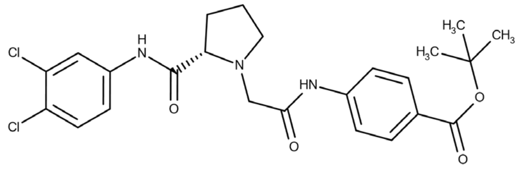

BP96. tert-butyl (S)-4-(2-(2-((3,4-dichlorophenyl)carbamoyl)pyrrolidin-1-yl)acetamido)benzoate was prepared as outlined in general procedure B using **BP91** and **intermediate 7** on a 0.371mmol scale. ^{1}H NMR (400 MHz, Chloroform-*d*) δ 10.05 (s, 1H), 8.86 (s, 1H), 8.00 – 7.93 (m, 3H), 7.67 (d, *J* = 8.2 Hz, 2H), 7.52 (d, *J* = 8.7, 2.4 Hz, 1H), 7.36 (d, *J* = 8.8 Hz, 1H), 3.61 (d, *J* = 16.3 Hz, 1H), 3.49 – 3.42 (m, 1H), 3.41 – 3.33 (m, 1H), 2.65 (q, *J* = 8.3 Hz, 1H), 2.36 – 2.22 (m, 1H), 2.16 – 2.06 (m, 2H), 1.96 – 1.85 (m, 2H), 1.60 (s, 9H). ^{13}C NMR (101 MHz, Chloroform-*d*) δ 173.26, 169.62, 165.32, 141.37, 137.61, 132.56, 130.59, 121.33, 80.97, 68.57, 59.26, 55.05, 36.55, 32.94, 31.51, 28.22, 25.03. HPLC-MS t_R = 8.77 minutes, 99.7% purity [$C_{24}H_{27}Cl_2N_3O_4$ + H] = 492.1, found 492.1 (108.1mg, 92% yield).

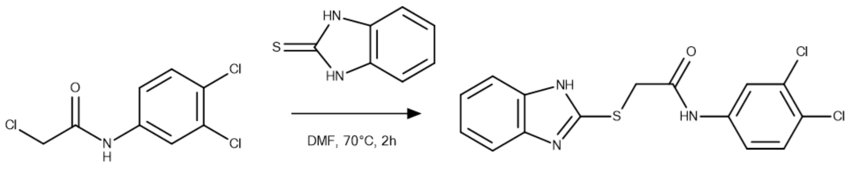

5

BP101

Scheme 16: Synthesis of **BP101**

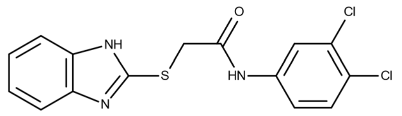

BP101. 2-chloro-N-(3,4-dichlorophenyl)acetamide (0.419mmol, 1 equiv.) was combined with 2-mercaptobenzimidazole (0.629mmol, 1.5 equiv.) in anhydrous DMF and stirred at 70˚C for 2h. 2-((1H-benzoimidazol-2-yl)thio)-N-(3,4-dichlorophenyl)acetamide was purified by flash chromatography using a mixture of hexane and ethyl acetate. ^{1}H NMR (400 MHz, DMSO) δ 12.69 (s, 1H), 10.80 (s, 1H), 7.99 (d, *J* = 2.2 Hz, 1H), 7.58 (d, *J* = 8.8 Hz, 1H), 7.52 – 7.40 (m, 3H), 7.14 (dt, *J* = 6.2, 3.7 Hz, 2H), 4.30 (s, 2H). ^{13}C NMR (101 MHz, DMSO) δ 167.19, 150.07, 139.39, 131.56, 131.27, 125.41, 122.00, 120.74, 119.61, 36.61. HPLC-MS t_R = 8.99 minutes, 92.7% purity [$C_{24}H_{27}Cl_2N_3O_4S$ + H] = 352.0, found 352.0 (31.2mg, 19% yield).

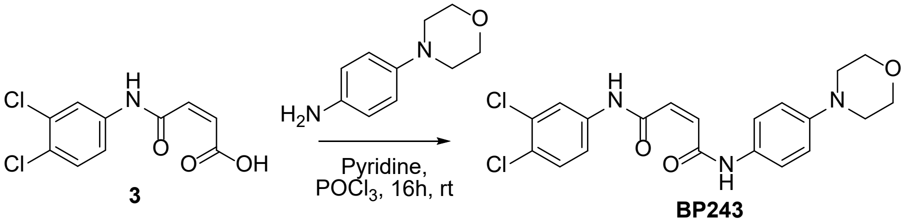

3

BP243

Scheme 17: Synthesis of **BP243**.

BP243. Intermediate **3** (0.385mmol, 1 equiv.) was placed in a round-bottom flask and sealed with a rubber septum. The flask was vented with N_2 gas. Pyridine (1.5mL) was injected into the flask and the solution was stirred. 2-methylthio aniline (0.385mmol, 1 equiv.) was added to the flask. Phosphoryl chloride (0.423mmol, 1.1 equiv.) was added to the flask in a dropwise manner and the reaction was allowed to stir for 16h at room temperature. The reaction was quenched in cold water and the precipitate was collected by gravity filtration. The precipitate was redissolved in methanol and purified by normal phase high performance liquid chromatography (5.4mg, 3% yield). ^{1}H NMR (400 MHz, DMSO) δ 10.79 (s, 1H), 10.39 (s, 1H), 8.09 (d, *J* = 2.3 Hz, 1H), 7.65 – 7.54 (m, 4H), 7.23 – 7.06 (m, 2H), 6.93 (d, *J* = 8.6 Hz, 2H), 3.73 (t, *J* = 4.6 Hz, 4H), 3.06 (t, *J* = 4.8 Hz, 4H). ^{13}C NMR (101 MHz, DMSO) δ 163.09, 161.63, 148.19, 139.33, 135.56, 133.08, 131.59, 131.37, 131.33, 125.82, 121.03, 120.88, 119.94, 115.85, 66.55, 49.18, 40.64, 40.43, 40.22, 40.01, 39.80, 39.60, 39.39. HPLC-MS t_R = 8.956min, 100% purity, [$C_{20}H_{19}Cl_2N_3O_3$ + H] = 420.1, found 420.1.

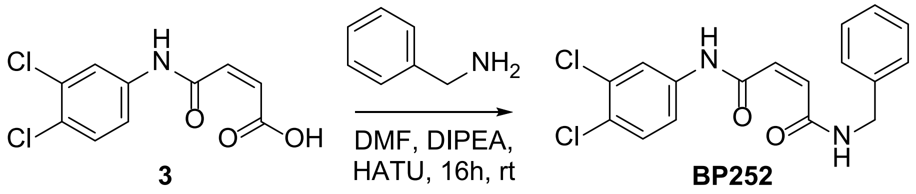

Scheme 8: Synthesis of **BP252**.

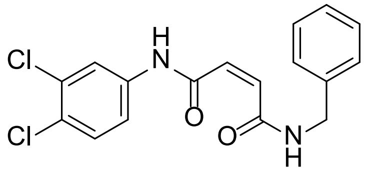

BP252. *N*[1]-benzyl-*N*[4]-(3,4-dichlorophenyl)maleamide was prepared as outlined in General Procedure D using benzylamine on a 0.327mmol scale (22.7mg, 18% yield). ^{1}H NMR (400 MHz, DMSO) δ 11.12 (s, 1H), 8.98 (t, *J* = 6.0 Hz, 1H), 8.03 (d, *J* = 2.5 Hz, 1H), 7.62 – 7.45 (m, 2H), 7.35 – 7.22 (m, 5H), 6.35 (d, *J* = 1.9 Hz, 2H), 4.35 (d, *J* = 5.9 Hz, 2H). ^{13}C NMR (101 MHz, DMSO) δ 165.01, 164.62, 139.47, 139.24, 132.88, 131.49, 131.18, 128.77, 127.94, 127.41, 125.30, 120.90, 119.80, 42.73. HPLC-MS t_R = 7.060min, 99.8% purity, [$C_{17}H_{14}Cl_2N_2O_2$ + H] = 349.0, found 349.0.

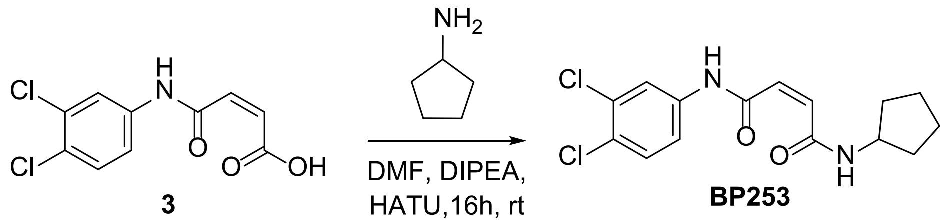

Scheme 9: Synthesis of **BP253**.

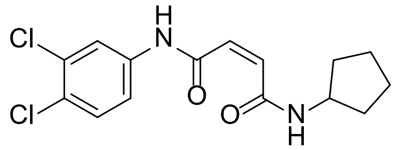

BP253. *N*[1]-cyclopentyl- *N*[4]-(3,4-dichlorophenyl)maleamide was prepared as outlined in General Procedure D using cyclopentylamine and having been further purified by normal phase high performance liquid chromatography on a 0.779mmol scale (111.16mg, 44% yield). ^{1}H NMR (400 MHz, DMSO) δ 11.49 (s, 1H), 8.56 (d, *J* = 7.3 Hz, 1H), 8.00 (d, *J* = 2.4 Hz, 1H), 7.61 – 7.42 (m, 2H), 6.27 (d, *J* = 2.1 Hz, 2H), 4.05 (q, *J* = 6.6 Hz, 1H), 1.82 (dq, *J* = 12.9, 6.3 Hz, 2H), 1.64 (t, *J* = 7.7 Hz, 2H), 1.56 – 1.36 (m, 4H). ^{13}C NMR (101 MHz, DMSO) δ 164.01, 163.96, 138.98, 132.65, 131.02, 130.81, 130.72, 124.80, 120.38, 119.31, 50.46, 32.09, 23.47. HPLC-MS t_R = 9.522min, 99.5% purity, [$C_{15}H_{16}Cl_2N_2O_2$ + H] = 327.0, found 327.0.

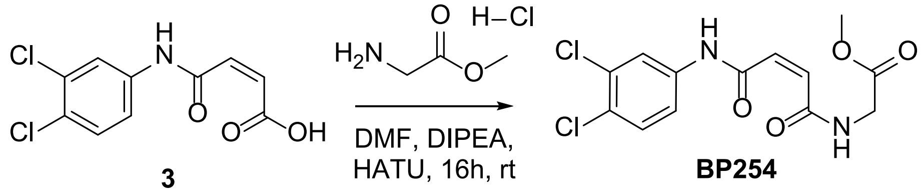

Scheme 10: Synthesis of **BP254**

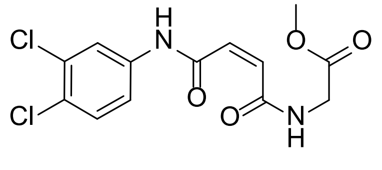

BP254. Methyl (4-((3,4-dichlorophenyl)amino)-4-oxobut-2-enoyl)glycinate was prepared as outlined in General Procedure D using methyl glycinate hydrochloride on a 1.083mmol scale (72.8mg, 20% yield). ^{1}H NMR (400 MHz, DMSO) δ 10.92 (s, 1H), 8.96 (t, *J* = 5.9 Hz, 1H), 8.01 (d, *J* = 2.4 Hz, 1H), 7.57 (d, *J* = 8.8 Hz, 1H), 7.47 (dd, *J* = 8.7, 2.4 Hz, 1H), 3.95 (d, *J* = 5.9 Hz, 2H), 3.64 (s, 3H). ^{13}C NMR (101 MHz, DMSO) δ 169.98, 164.69, 164.31, 138.92, 133.22,

131.00, 130.69, 129.52, 124.87, 120.47, 119.37, 51.79, 40.64. HPLC-MS t_R = 7.987min, 88.9% purity, [$C_{13}H_{12}Cl_2N_2O_4$ - H] = 329.0, found 329.0.

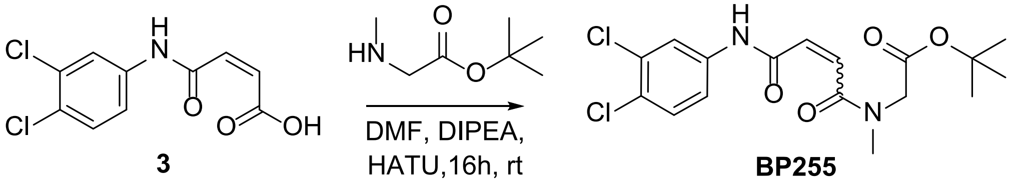

Scheme 11: Synthesis of **BP255**.

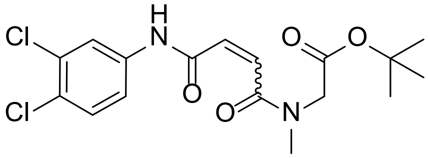

BP255. *N*-(4-((3,4-dichlorophenyl)amino)-4-oxobut-2-enoyl) tert butyl sarcosine was prepared as outlined in General Procedure D using tert butyl sarcosine on a 2.214mmol scale (304.7mg, 36% yield). ^{1}H NMR (400 MHz, DMSO) δ 10.56 (d, *J* = 3.9 Hz, 2H), 8.04 (t, *J* = 2.6 Hz, 2H), 7.57 (d, *J* = 8.7 Hz, 2H), 7.46 (dd, *J* = 8.9, 2.4 Hz, 2H), 6.64 (d, *J* = 11.8 Hz, 1H), 6.25 (d, *J* = 11.8 Hz, 1H), 4.03 (s, 2H), 2.94 (s, 3H), 1.42 (s, 9H). ^{13}C NMR (101 MHz, DMSO) δ 168.53, 167.87, 163.13, 139.31, 135.72, 131.17, 126.71, 125.48, 120.84, 119.74, 81.39, 52.42, 49.01, 38.69, 28.16. HPLC-MS t_R = 9.036min, 99.4% purity, [$C_{17}H_{20}Cl_2N_2O_4$ - H] = 385.0, found 385.0.

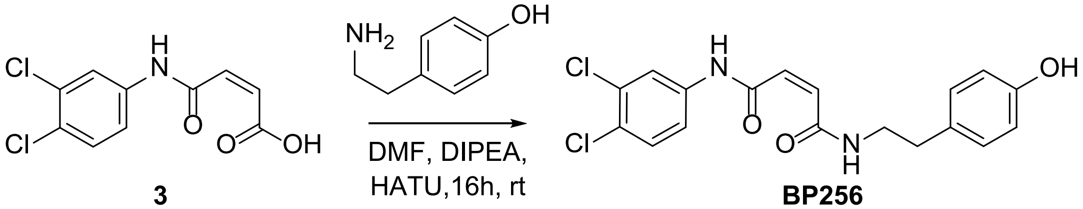

Scheme 12: Synthesis of **BP256**.

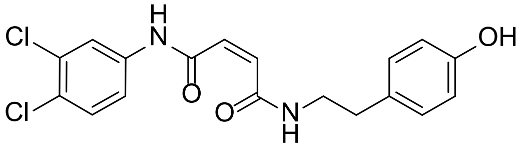

BP256. *N*^{1}-(3,4-dichlorophenyl)-*N*^{4}-(4-hydroxyphenethyl)maleamide was prepared as outlined in General Procedure D using 4-(2-aminoethyl)phenol on a 0.495mmol scale (39.4mg, 21% yield).

^{1}H NMR (400 MHz, DMSO) δ 11.36 (s, 1H), 9.15 (s, 1H), 8.60 (d, *J* = 5.9 Hz, 1H), 8.01 (d, *J* = 2.3 Hz, 1H), 7.57 (d, *J* = 8.8 Hz, 1H), 7.48 (dd, *J* = 8.8, 2.5 Hz, 1H), 7.01 (d, *J* = 8.3 Hz, 2H), 6.70 – 6.63 (m, 2H), 6.33 – 6.22 (m, 2H), 3.31 – 3.26 (m, 2H), 2.64 (t, *J* = 7.4 Hz, 2H). ^{13}C NMR (101 MHz, DMSO) δ 164.97, 164.47, 156.16, 139.43, 132.97, 131.51, 131.35, 131.19, 129.95, 129.73, 125.32, 120.89, 119.81, 115.59, 41.22, 34.44. HPLC-MS t_R = 8.687min, 97.3% purity, [$C_{18}H_{16}Cl_2N_2O_3$ - H] = 377.1, found 377.0.

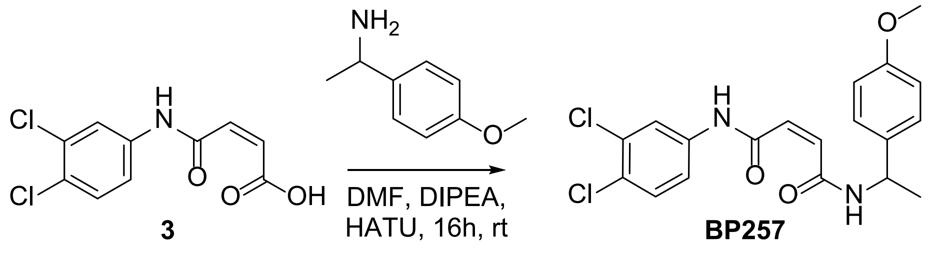

Scheme 13: Synthesis of **BP257**.

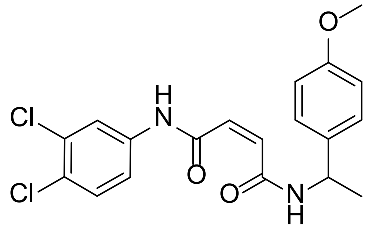

BP257. *N*[1]-(3,4-dichlorophenyl)-*N*[4]-(1-(4-methoxyphenethyl))maleamide was prepared as outlined in General Procedure D using 1-(4-methoxyphenyl)ethan-1-amine on a 0.603mmol scale (76.8mg, 34% yield). ^{1}H NMR (400 MHz, DMSO) δ 11.20 (s, 1H), 8.90 (d, *J* = 8.1 Hz, 1H), 8.00 (d, *J* = 2.4 Hz, 1H), 7.57 (d, *J* = 8.8 Hz, 1H), 7.46 (dd, *J* = 8.7, 2.4 Hz, 1H), 7.25 (d, *J* = 8.3 Hz, 2H), 6.87 (d, *J* = 8.3 Hz, 2H), 6.37 – 6.25 (m, 2H), 4.93 (p, *J* = 7.1 Hz, 1H), 3.72 (s, 3H), 1.36 (d, *J* = 7.0 Hz, 3H). ^{13}C NMR (101 MHz, DMSO) δ 164.24, 163.38, 158.15, 138.98, 135.84, 132.81, 131.00, 130.70, 130.43, 127.28, 124.79, 120.41, 119.31, 113.63, 55.05, 47.42, 22.16. HPLC-MS t_R = 9.395min, 97.5% purity, [$C_{19}H_{18}Cl_2N_2O_3$ - H] = 391.1, found 391.0.

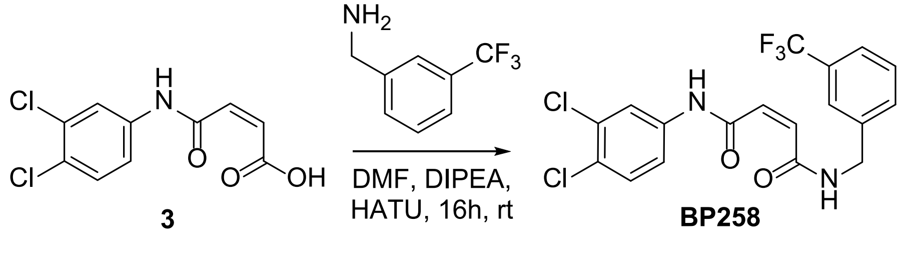

Scheme 14: Synthesis of **BP258**.

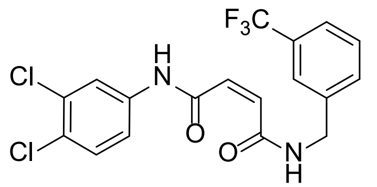

BP258. *N*[1]-(3,4-dichlorophenyl)-*N*[4]-((3-trifluoromethyl)benzyl)maleamide was prepared as outlined in General Procedure D using 3-trifluoromethyl benzylamine on a 0.684mmol scale (74.4mg, 26% yield). ^{1}H NMR (400 MHz, DMSO) δ 10.89 (s, 1H), 8.96 (t, *J* = 6.0 Hz, 1H), 8.07 (d, *J* = 2.4 Hz, 1H), 7.77 (s, 1H), 7.64 – 7.52 (m, 4H), 7.47 (dd, *J* = 8.8, 2.4 Hz, 1H), 6.37 (q, *J* = 12.1 Hz, 2H), 4.44 (d, *J* = 5.9 Hz, 2H). ^{13}C NMR (101 MHz, DMSO) δ 165.15, 163.81, 140.56, 139.00, 131.96, 131.47, 131.06, 130.92, 130.64, 129.21, 124.87, 120.39, 119.25, 41.68. HPLC-MS t_R = 9.743min, 98.6% purity, [$C_{18}H_{13}Cl_2F_3N_2O_3$ - H] = 415.0, found 415.0.

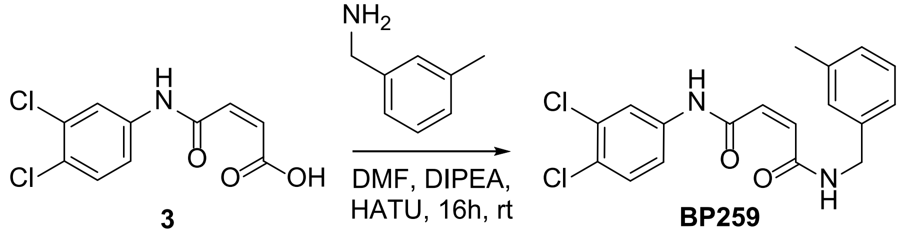

Scheme 15: Synthesis of **BP259**.

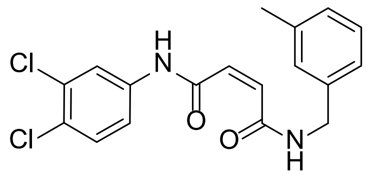

BP259. *N*[1]-(3,4-dichlorophenyl)-*N*[4]-((3-methyl)benzyl)maleamide was prepared as outlined in General Procedure D using 3-trifluoromethyl benzylamine on a 0.641mmol scale (32.0mg, 14% yield). ^{1}H NMR (400 MHz, DMSO) δ 11.12 (s, 1H), 8.93 (t, *J* = 5.9 Hz, 1H), 8.05 (d, *J* = 2.4 Hz, 1H), 7.61 – 7.54 (m, 1H), 7.48 (dd, *J* = 8.8, 2.4 Hz, 1H), 7.19 (dd, *J* = 17.8, 10.3 Hz, 2H), 7.07 (t, *J* = 9.0 Hz, 2H), 6.35 (s, 2H), 4.31 (d, *J* = 5.8 Hz, 2H), 2.28 (s, 3H). ^{13}C NMR (101 MHz, DMSO) δ 164.54, 164.11, 139.01, 138.67, 137.39, 132.07, 131.01, 130.96, 130.71, 128.18, 128.08, 127.53, 124.54, 120.39, 119.30, 42.17, 20.98. HPLC-MS t_R = 9.672min, 98.3% purity, [$C_{18}H_{16}Cl_2N_2O_2$ - H] = 361.1, found 361.0.

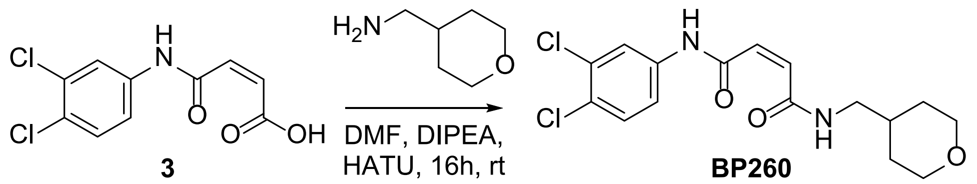

Scheme 16: Synthesis of **BP260**.

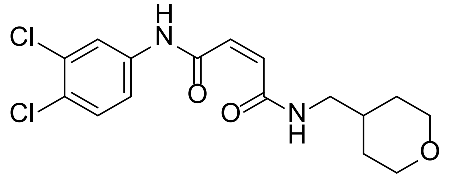

BP260. *N*[1]-(3,4-dichlorophenyl)-*N*[4]-((4-tetrahydropyranyl)methyl)maleamide was prepared as outlined in General Procedure D using 4-aminomethyltetrahydropyran on a 0.733mmol scale (93.1mg, 36% yield). ^{1}H NMR (400 MHz, $CDCl_3$) δ 12.42 (s, 1H), 7.93 (d, J = 2.4 Hz, 1H), 7.51 (dd, J = 8.7, 2.4 Hz, 1H), 7.37 (d, J = 8.7 Hz, 1H), 6.93 (s, 1H), 6.27 (d, J = 13.5 Hz, 1H), 6.16 (d, J = 13.5 Hz, 1H), 4.03 – 3.94 (m, 2H), 3.38 (td, J = 11.7, 2.0 Hz, 2H), 3.28 (t, J = 6.5 Hz, 2H), 1.84 (ddt, J = 11.6, 8.1, 4.2 Hz, 1H), 1.65 (d, J = 12.9 Hz, 2H), 1.36 (qd, J = 12.2, 4.5 Hz, 2H). ^{13}C NMR (101 MHz, DMSO) δ 165.19, 164.49, 139.44, 132.66, 131.52, 131.50, 131.19, 125.30, 120.86, 119.77, 67.18, 44.93, 35.10, 30.86. HPLC-MS t_R = 8.754, 97.5% purity, [$C_{16}H_{18}Cl_2N_2O_3$ + H] = 357.1, found 357.0.

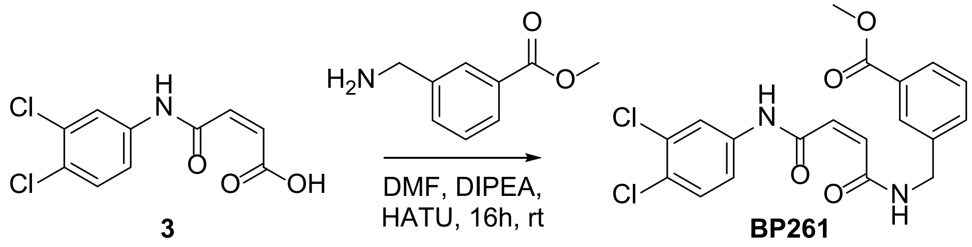

Scheme 17: Synthesis of **BP261**.

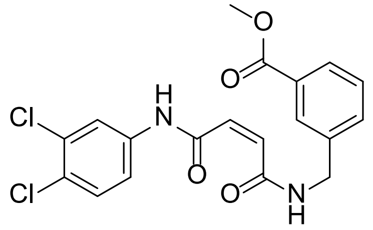

BP261. Methyl 3-((4-((3,4-dichlorophenyl)amino)-4-oxobut-2-enamido)methyl)benzoate was prepared as outlined in General Procedure D using methyl 3-(aminomethyl)benzoate on a 0.742mmol scale (94.2mg, 31% yield). ^{1}H NMR (400 MHz, DMSO) δ 10.99 (s, 1H), 9.02 (t, *J* = 6.0 Hz, 1H), 8.02 (d, *J* = 2.4 Hz, 1H), 7.92 (s, 1H), 7.85 (dt, *J* = 7.7, 1.5 Hz, 1H), 7.58 (dd, *J* = 12.0, 8.3 Hz, 2H), 7.48 (ddd, *J* = 7.8, 4.6, 2.2 Hz, 2H), 6.40 – 6.32 (m, 2H), 4.42 (d, *J* = 5.9 Hz, 2H), 3.84 (s, 3H). ^{13}C NMR (101 MHz, DMSO) δ 166.18, 164.74, 164.08, 139.68, 138.98, 132.46, 132.09, 131.03, 130.90, 130.68, 129.72, 128.77, 128.12, 127.78, 124.85, 120.44, 119.32, 52.13, 41.89. HPLC-MS t_R = 8.754, 97.8% purity, [$C_{19}H_{16}Cl_2N_2O_4$ + H] = 407.1, found 407.0.

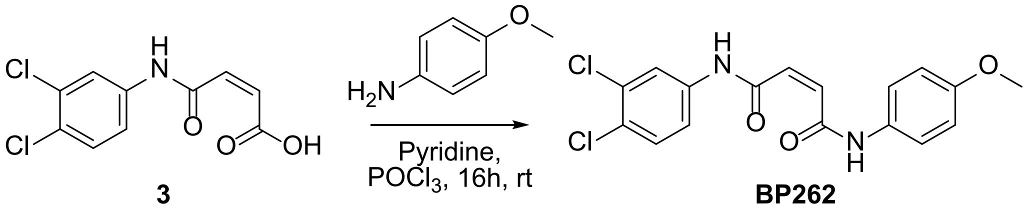

Scheme 18: Synthesis of **BP262**.

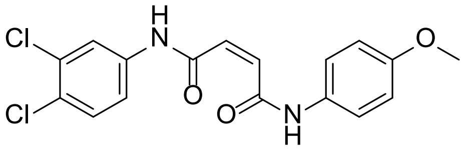

BP262. Intermediate **3** (0.605mmol, 1 equiv.) was placed in a round-bottom flask and sealed with a rubber septum. The flask was vented with N_2 gas. Pyridine (1.5mL) was injected into the flask and the solution was stirred. 4-methoxy aniline (0.605mmol, 1 equiv.) was added to the flask. Phosphoryl chloride (0.666mmol, 1.1 equiv.) was added to the flask in a dropwise manner and the reaction was allowed to stir for 16h at room temperature. The reaction was quenched in cold water and extracted by ethyl acetate. The product was initially purified by automated flash chromatography using a mixture of hexane and ethyl acetate. The product further purified by normal phase high performance liquid chromatography (5.3mg, 2% yield). ^{1}H NMR (400 MHz, DMSO) δ 11.27 (s, 1H), 10.68 (s, 1H), 8.16 (s, 1H), 7.63 (dd, *J* = 24.3, 8.9 Hz, 4H), 7.23 (d, *J* = 3.9 Hz, 2H), 6.92 (d, *J* = 8.6 Hz, 2H), 3.73 (s, 3H). ^{13}C NMR (101 MHz, DMSO) δ 164.38, 162.61, 155.54, 139.03, 131.97, 131.84, 131.21, 131.00, 130.72, 124.83, 120.95, 120.43, 119.36, 113.92, 55.18. HPLC-MS t_R = 9.715min, 99.2% purity, [$C_{17}H_{14}Cl_2N_2O_3$ - H] = 363.0, found 363.1.

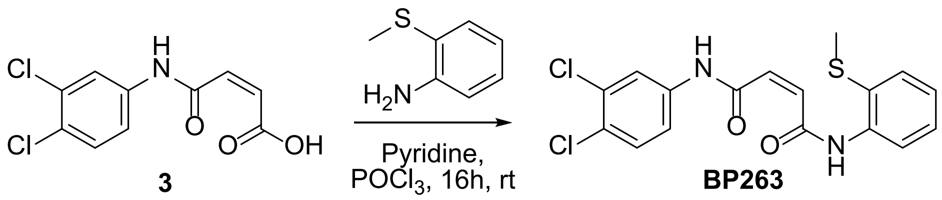

Scheme 19: Synthesis of **BP263**.

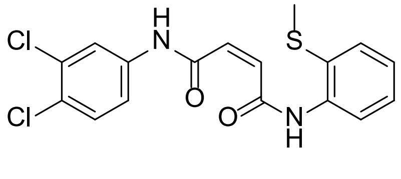

BP263. Intermediate **3** (0.484mmol, 1 equiv.) was placed in a round-bottom flask and sealed with a rubber septum. The flask was vented with N_2 gas. Pyridine (1.5mL) was injected into the flask and the solution was stirred. 2-methylthio aniline (0.484mmol, 1 equiv.) was added to the flask. Phosphoryl chloride (0.532mmol, 1.1 equiv.) was added to the flask in a dropwise manner and the reaction was allowed to stir for 16h at room temperature. The reaction was quenched in cold water and extracted by ethyl acetate. The product was initially purified by automated flash chromatography using a mixture of hexane and ethyl acetate. The product further purified by normal phase high performance liquid chromatography (2.4mg, 1% yield). ^{1}H NMR (400 MHz, MeOD) δ 7.98 (s, 1H), 7.70 (s, 1H), 7.54 – 7.40 (m, 3H), 7.24 (dd, *J* = 5.9, 3.5 Hz, 2H), 6.56 (d, *J* = 12.6 Hz, 1H), 6.45 (d, *J* = 12.6 Hz, 1H), 2.43 (s, 3H). ^{13}C NMR (101 MHz, DMSO) δ 163.00, 162.87, 139.30, 134.82, 134.26, 134.02, 131.60, 131.32, 127.28, 126.75, 125.88, 125.72, 121.07, 119.96, 15.63. HPLC-MS t_R = 10.149, 98.4% purity, $[C_{17}H_{14}Cl_2N_2O_2S + H]$ = 381.0, found 381.0.

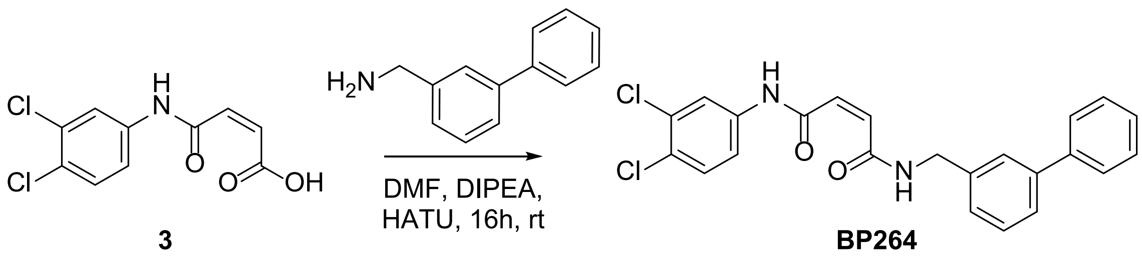

Scheme 20: Synthesis of **BP264**.

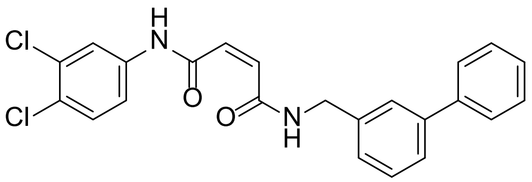

BP264. *N*[1]-([1,1'-biphenyl]-3-ylmethyl)-*N*[4]-(3,4-dichlorophenyl)maleamide was prepared as outlined in General Procedure D using [1,1'-biphenyl]-3-ylmethanamine on a 0.512mmol scale. The product was further purified by normal phase high performance liquid chromatography (10.0mg, 5% yield). ^{1}H NMR (400 MHz, Acetone) δ 12.75 (s, 1H), 8.81 (s, 1H), 8.11 (s, 1H), 7.70 – 7.35 (m, 11H), 6.50 (d, *J* = 13.3 Hz, 1H), 6.27 (d, *J* = 13.3 Hz, 1H), 4.65 (d, *J* = 4.8 Hz, 2H). ^{13}C NMR (101 MHz, Acetone) δ 206.20, 166.44, 163.51, 142.16, 141.59, 139.99, 139.67, 136.54, 136.28, 132.75, 131.51, 131.40, 129.98, 129.72, 128.30, 127.75, 127.67, 127.30, 126.75, 121.68, 120.14. HPLC-MS t_R = 10.836, 97.5% purity, [$C_{23}H_{18}Cl_2N_2O_2$ - H] = 423.1, found 423.0.

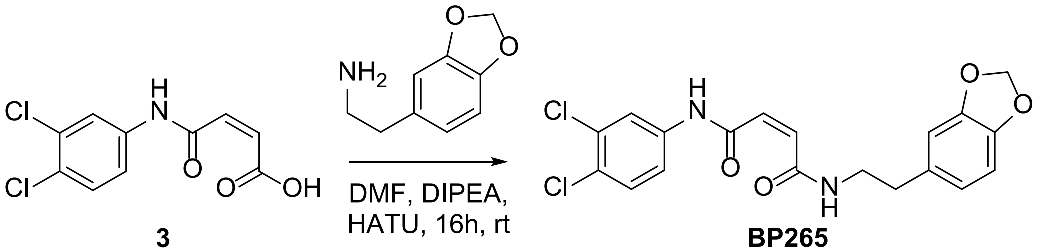

3

BP265

Scheme 21: Synthesis of **BP265**.

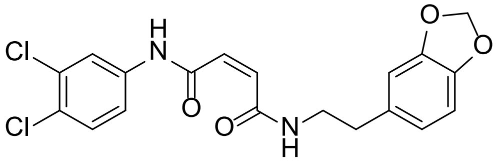

BP265. *N*[1]-(3,4-methylenedioxyphenethyl)-*N*[4]-(3,4-dichlorophenyl)maleamide was prepared as outlined in General Procedure D using 3,4-methylenedioxyphenethylamine on a 0.444mmol scale (62.5mg, 35% yield). ^{1}H NMR (400 MHz, Acetone) δ 12.94 (s, 1H), 8.39 (s, 1H), 8.10 (s, 1H), 7.52 (s, 2H), 6.80 (s, 1H), 6.74 (q, *J* = 8.0 Hz, 2H), 6.39 (d, *J* = 13.4 Hz, 1H), 6.20 (d, *J* = 13.3 Hz, 1H), 5.93 (s, 2H), 3.57 (q, *J* = 6.8 Hz, 2H), 2.82 (t, *J* = 7.2 Hz, 2H). ^{13}C NMR (101 MHz, DMSO) δ 164.56, 163.99, 147.19, 145.55, 138.95, 133.05, 132.41, 131.04, 130.95, 130.72, 124.87, 121.55, 120.43, 119.34, 109.06, 108.09, 100.65, 40.57, 34.44. HPLC-MS t_R = 10.037min, 95.5% purity, [$C_{19}H_{16}Cl_2N_2O_4$ - H] = 405.1, found 405.0.

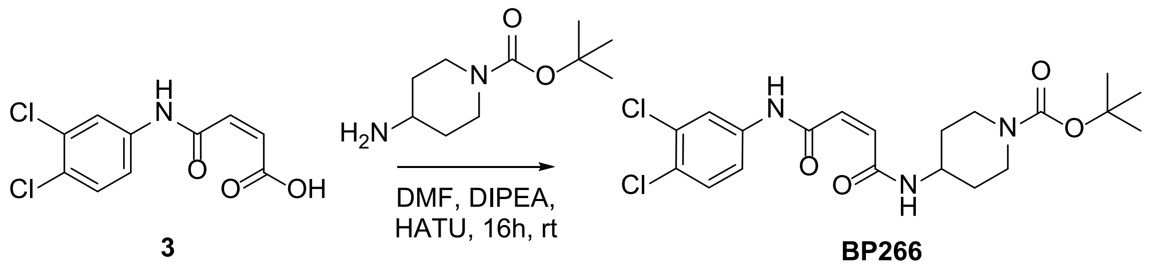

Scheme 22: Synthesis of **BP266**.

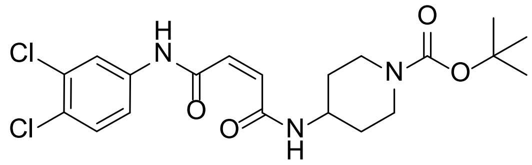

BP266. Tert-butyl 4-(4-((3,4-dichlorophenyl)amino)-4-oxobut-2-enamido)piperidine-1-carboxylate was prepared as outlined in General Procedure D using tert-butyl 4-aminopiperidine-1-carboxylate on a 0.393mmol scale (71.2mg, 41% yield). ^{1}H NMR (400 MHz, $CDCl_3$) δ 12.58 (s, 1H), 7.89 (d, *J* = 2.4 Hz, 1H), 7.49 (dd, *J* = 8.7, 2.4 Hz, 1H), 7.33 (d, *J* = 8.7 Hz, 1H), 6.24 – 6.14 (m, 2H), 4.04 (s, 2H), 3.99 – 3.93 (m, 1H), 2.90 (t, *J* = 12.7 Hz, 2H), 1.92 (dd, *J* = 13.0, 3.8 Hz, 2H), 1.43 (s, 9H), 1.42 – 1.37 (m, 2H). ^{13}C NMR (101 MHz, $CDCl_3$) δ 164.73, 162.59, 154.73, 137.70, 136.51, 132.65, 130.48, 130.44, 127.55, 121.76, 119.49, 80.02, 47.50, 38.68, 31.45, 28.44. HPLC-MS t_R = 10.214min, 98.6% purity, [$C_{20}H_{25}Cl_2N_3O_4$ - H] = 440.1, found 440.1.

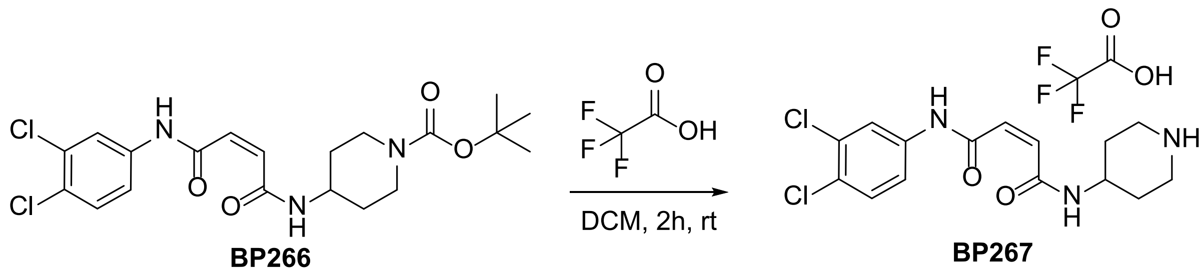

Scheme 23: Synthesis of **BP267**.

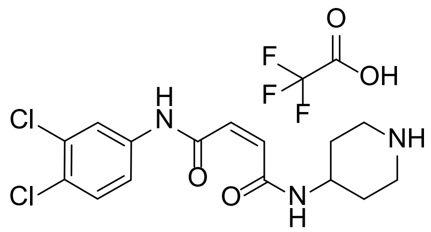

BP267. N^1-(3,4-dichlorophenyl)-N^4-(piperidin-4-yl)maleamide 2,2,2-trifluoroacetate (0.115mmol, 1 equiv.) was dissolved in dichloromethane in a round-bottom flask. Trifluoroacetic acid (6.230mmol, 57 equiv.) was added dropwise to the stirred solution and the reaction was stirred for 2h. The reaction was concentrated under reduced pressure. N^1-(3,4-dichlorophenyl)-N^4-(piperidin-4-yl)maleamide was resuspended in dichloromethane and solvent was evaporated under reduced pressure, repeated a total of 6 times (25.6mg, 65% yield). ^{1}H NMR (400 MHz, MeOD) δ 7.96 (d, J = 2.2 Hz, 1H), 7.50 – 7.39 (m, 2H), 6.33 (d, J = 1.8 Hz, 2H), 4.07 – 4.01 (m, 1H), 3.43 (dt, J = 13.3, 4.2 Hz, 2H), 3.18 – 3.08 (m, 2H), 2.17 (dd, J = 14.2, 4.1 Hz, 2H), 1.80 (ddt, J = 14.4, 10.6, 5.5 Hz, 2H). ^{13}C NMR (101 MHz, MeOD) δ 166.61, 164.58, 138.78, 132.63, 132.57, 132.37, 130.85, 127.41, 121.83, 119.92, 78.70, 44.79, 43.08, 38.21, 28.29. HPLC-MS t_R = 7.118min, 98.9% purity, [$C_{15}H_{17}Cl_2N_3O_2$ - H] = 340.1, found 340.0.

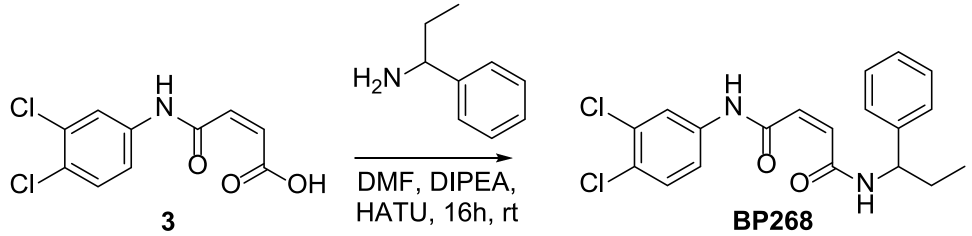

3

BP268

Scheme 24: Synthesis of **BP268**.

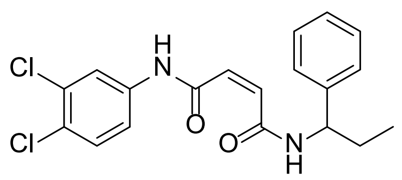

BP268. N^1-(3,4-dichlorophenyl)-N^4-(1-phenylpropyl)maleamide was prepared as outlined in General Procedure D using 1-phenylpropan-1-amine on a 0.327mmol scale (22.7mg, 18% yield). ^{1}H NMR (400 MHz, MeOD) δ 7.95 (d, J = 2.1 Hz, 1H), 7.46 – 7.43 (m, 2H), 7.35 – 7.30 (m, 4H), 7.24 (tq, J = 5.7, 2.7 Hz, 1H), 6.39 – 6.26 (m, 2H), 4.84 (t, J = 7.4 Hz, 1H), 1.84 (pt, J = 10.3, 5.4 Hz, 2H), 0.93 (t, J = 7.3 Hz, 3H). ^{13}C NMR (101 MHz, MeOD) δ 166.50, 165.59, 143.48, 139.56, 134.08, 133.38, 132.62, 131.58, 129.52, 128.28, 127.79, 122.60, 120.68, 56.88, 30.38, 11.26. HPLC-MS t_R = 11.060min, 95.1% purity, [$C_{19}H_{18}Cl_2N_2O_2$ - H] = 375.1, found 375.1.

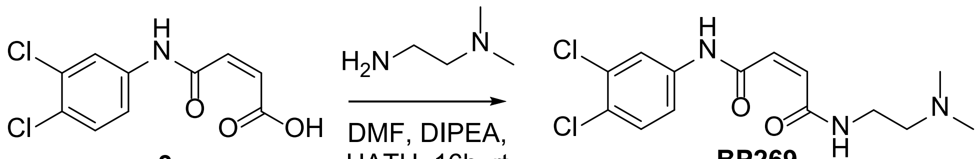

3 **BP269**

Scheme 25: Synthesis of **BP269**.

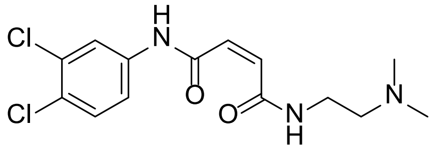

BP269. *N*[1]-(3,4-dichlorophenyl)-*N*[4]-(2-(dimethylamino)ethyl)maleamide was prepared as outlined in General Procedure D using N[1],N[1]-dimethylethane-1,2-diamine on a mmol scale (5.1mg, % yield). ^{1}H NMR (400 MHz, DMSO) δ 10.85 (s, 1H), 9.68 (s, 1H), 8.84 (s, 1H), 8.10 (s, 1H), 7.72 – 7.49 (m, 2H), 7.06 (d, *J* = 15.2 Hz, 1H), 6.98 (d, *J* = 15.0 Hz, 1H), 3.54 (d, *J* = 6.3 Hz, 2H), 3.36 (s, 6H), 3.20 (t, *J* = 6.1 Hz, 2H). ^{13}C NMR (101 MHz, DMSO) δ 163.26, 162.72, 138.88, 134.66, 132.06, 131.08, 130.79, 125.26, 120.50, 119.40, 57.88, 45.04, 36.94. HPLC-MS t_R = 7.426min, 99.1% purity, [$C_{14}H_{17}Cl_2N_3O_2$ + H] = 330.1, found 330.0.

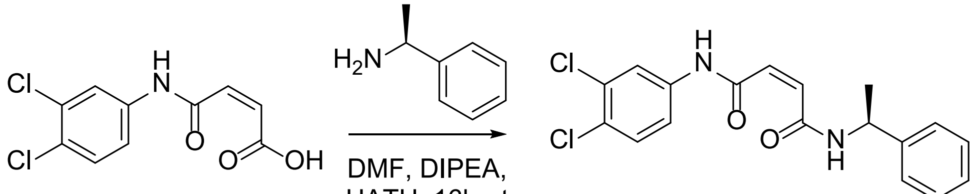

3 **BP270**

Scheme 26: Synthesis of **BP270**.

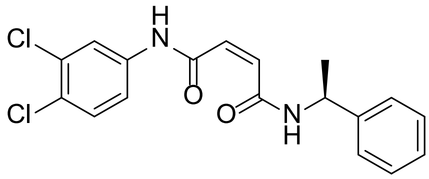

BP270. *N*[1]-(3,4-dichlorophenyl)-*N*[4]-((*S*)-1-phenyethyl)maleamide was prepared as outlined in General Procedure D using (*S*)-1-phenylethan-1-amine on a 0.246mmol scale (34.8mg, 39% yield). ^{1}H NMR (400 MHz, Acetone) δ 12.81 (s, 1H), 8.75 (s, 1H), 8.08 (d, *J* = 2.3 Hz, 1H), 7.50

(s, 2H), 7.42 (d, *J* = 7.6 Hz, 2H), 7.37 – 7.31 (m, 2H), 7.28 – 7.23 (m, 1H), 6.45 (dd, *J* = 13.4, 2.3 Hz, 1H), 6.23 (dd, *J* = 13.3, 2.4 Hz, 1H), 5.19 (q, *J* = 7.5 Hz, 1H), 1.53 (dd, *J* = 7.0, 2.2 Hz, 3H). ^{13}C NMR (101 MHz, DMSO) δ 164.25, 163.48, 143.95, 138.97, 132.73, 130.98, 130.69, 130.38, 128.26, 126.77, 126.08, 124.77, 120.39, 119.30, 48.01, 22.22. HPLC-MS t_R =10.608min, 96.4% purity, [$C_{18}H_{16}Cl_2N_2O_2$ - H] = 361.1, found 361.0.

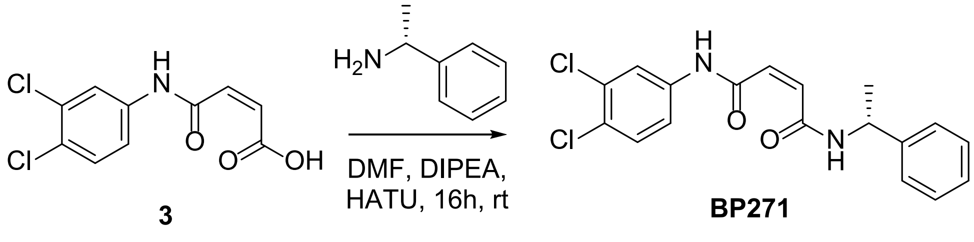

Scheme 27: Synthesis of **BP271**.

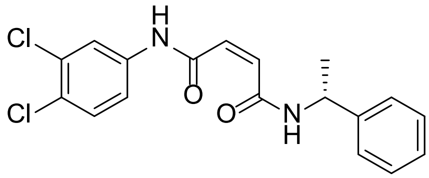

BP271. *N*[1]-(3,4-dichlorophenyl)-*N*[4]-((*R*)-1-phenyethyl)maleamide was prepared as outlined in General Procedure D using (*R*)-1-phenylethan-1-amine on a 0.246mmol scale (34.8mg, 39% yield). ^{1}H NMR (400 MHz, Acetone) δ 12.82 (s, 1H), 8.77 (s, 1H), 8.08 (d, *J* = 1.9 Hz, 1H), 7.50 (d, *J* = 2.4 Hz, 2H), 7.42 (d, *J* = 7.3 Hz, 2H), 7.35 (t, *J* = 7.5 Hz, 2H), 7.26 (t, *J* = 7.3 Hz, 1H), 6.46 (d, *J* = 13.4 Hz, 1H), 6.23 (d, *J* = 13.4 Hz, 1H), 5.20 (p, *J* = 7.2 Hz, 1H), 1.53 (d, *J* = 7.0 Hz, 3H). ^{13}C NMR (101 MHz, DMSO) δ 164.25, 163.49, 143.96, 138.98, 132.76, 131.00, 130.69, 130.40, 128.27, 126.78, 126.08, 124.78, 120.40, 119.30, 48.03, 22.22. HPLC-MS t_R =10.310min, 99.1% purity, [$C_{18}H_{16}Cl_2N_2O_2$ + H] = 363.1, found 363.1.

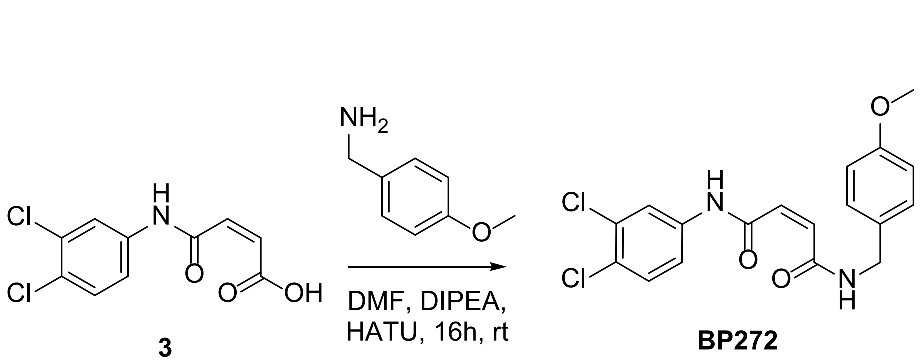

Scheme 28: Synthesis of **BP272**.

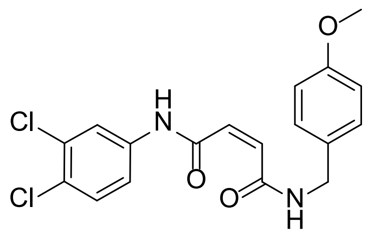

BP272. N^1-(3,4-dichlorophenyl)-N^4-(4-methoxybenzyl)maleamide was prepared as outlined in General Procedure D using 4-methoxy benzylamine on a 0.246mmol scale. The product was further purified by normal phase high performance liquid chromatography (10.0mg, 8% yield). ^{1}H NMR (400 MHz, Acetone) δ 12.88 (s, 1H), 8.71 (s, 1H), 8.11 (s, 1H), 7.52 (s, 2H), 7.29 (d, *J* = 8.6 Hz, 2H), 6.93 – 6.84 (m, 2H), 6.46 (d, *J* = 13.4 Hz, 1H), 6.24 (d, *J* = 13.3 Hz, 1H), 4.48 (d, *J* = 3.5 Hz, 2H), 3.77 (s, 3H). ^{13}C NMR (101 MHz, DMSO) δ 164.37, 164.15, 158.33, 139.00, 132.47, 131.00, 130.71, 130.65, 130.63, 128.85, 124.80, 120.41, 119.32, 113.70, 55.06, 41.72. HPLC-MS t_R =10.310min, 99.5% purity, [$C_{18}H_{16}Cl_2N_2O_3$ - H] = 377.1, found 377.0.

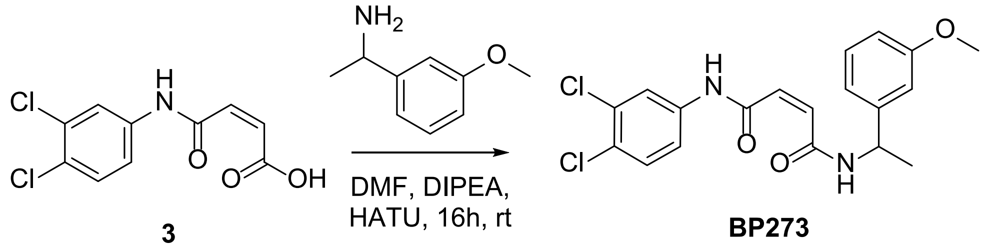

3 BP273

Scheme 29: Synthesis of **BP273**.

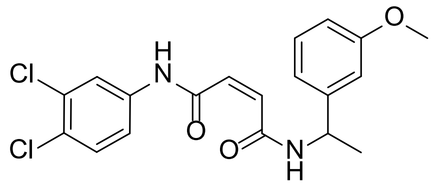

BP273. N^1-(3,4-dichlorophenyl)-N^4-(1-(3-methoxyphenyl)ethyl)maleamide was prepared as outlined in General Procedure D using (3-methoxyphenyl)ethan-1-amine on a 0.340mmol scale (46.2mg, 35% yield). ^{1}H NMR (400 MHz, DMSO) δ 11.10 (s, 1H), 8.92 (d, *J* = 8.1 Hz, 1H), 8.01 (d, *J* = 2.4 Hz, 1H), 7.57 (d, *J* = 8.8 Hz, 1H), 7.46 (dd, *J* = 8.8, 2.4 Hz, 1H), 7.23 (t, *J* = 7.8 Hz, 1H), 6.94 – 6.85 (m, 2H), 6.80 (dd, *J* = 8.2, 2.6 Hz, 1H), 6.33 (d, *J* = 3.0 Hz, 2H), 4.95 (p, *J* = 7.1 Hz, 1H), 3.74 (s, 3H), 1.37 (d, *J* = 7.0 Hz, 3H). ^{13}C NMR (101 MHz, DMSO) δ 164.20, 163.55, 159.30, 145.67, 138.97, 132.45, 131.00, 130.69, 129.32, 124.81, 120.41, 119.31, 118.27, 112.07, 111.93, 54.98, 48.02, 22.33. HPLC-MS t_R =9.498min, 95.1% purity, [$C_{19}H_{18}Cl_2N_2O_3$ + H] = 393.1, found 393.1.

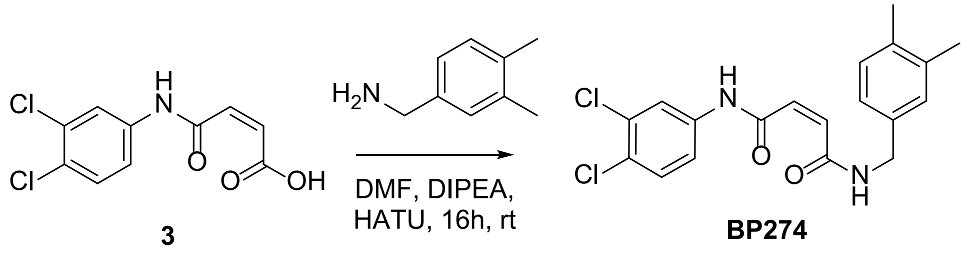

Scheme 30: Synthesis of **BP274**.

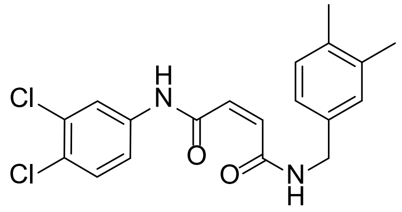

BP274. *N*[1]-(3,4-dichlorophenyl)-*N*[4]-(3,4-dimethylbenzyl)maleamide was prepared as outlined in General Procedure D using 3,4-dimethylbenzylamine on a 0.313mmol scale. The product was further purified by normal phase high performance liquid chromatography (38.0mg, 32% yield). ^{1}H NMR (400 MHz, Acetone) δ 12.90 (s, 1H), 8.67 (s, 1H), 8.12 (t, *J* = 1.5 Hz, 1H), 7.56 – 7.49 (m, 2H), 7.13 (s, 1H), 7.08 (d, *J* = 2.0 Hz, 2H), 6.47 (d, *J* = 13.3 Hz, 1H), 6.24 (d, *J* = 13.3 Hz, 1H), 4.47 (d, *J* = 5.8 Hz, 2H), 2.22 (s, 3H), 2.21 (s, 3H). ^{13}C NMR (101 MHz, DMSO) δ 164.45, 164.09, 139.00, 135.97, 134.68, 132.20, 131.02, 130.91, 130.70, 129.32, 128.71, 124.91, 124.81, 120.40, 119.30, 42.02, 40.15, 39.94, 39.73, 39.52, 39.31, 39.10, 38.89, 19.35, 18.99. HPLC-MS t_R = 9.903min, 95.6% purity, [$C_{19}H_{18}Cl_2N_2O_2$ + H] = 377.1, found 377.1.

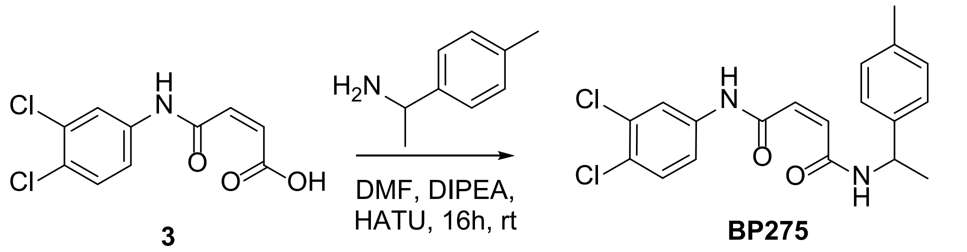

Scheme 31: Synthesis of **BP275**.

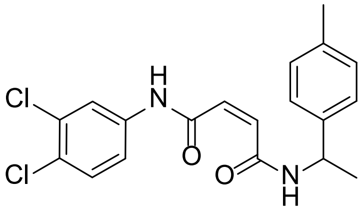

BP275. *N*[1]-(3,4-dichlorophenyl)-*N*[4]-(1-(*p*-tolyl)ethyl)maleamide was prepared as outlined in General Procedure D using 1-(*p*-tolyl)ethan-1-amine on a 0.340mmol scale (49.1mg, 39% yield). ^{1}H NMR (400 MHz, DMSO) δ 11.18 (s, 1H), 8.91 (d, *J* = 8.0 Hz, 1H), 8.00 (d, *J* = 2.4 Hz, 1H), 7.57 (d, *J* = 8.8 Hz, 1H), 7.46 (dd, *J* = 8.8, 2.4 Hz, 1H), 7.21 (d, *J* = 7.8 Hz, 2H), 7.12 (d, *J* = 7.7 Hz, 2H), 6.34 (d, *J* = 12.3 Hz, 1H), 6.30 (d, *J* = 12.4 Hz, 1H), 4.94 (p, *J* = 7.2 Hz, 1H), 2.26 (s, 3H), 1.36 (d, *J* = 7.0 Hz, 3H). ^{13}C NMR (101 MHz, DMSO) δ 164.21, 163.43, 140.89, 138.96, 135.83, 132.82, 130.99, 130.68, 130.40, 128.78, 126.01, 124.78, 120.40, 119.30, 47.76, 22.18, 20.60. HPLC-MS t_R = 10.174min, 95.3% purity, [$C_{19}H_{18}Cl_2N_2O_2$ - H] = 375.1, found 375.1.

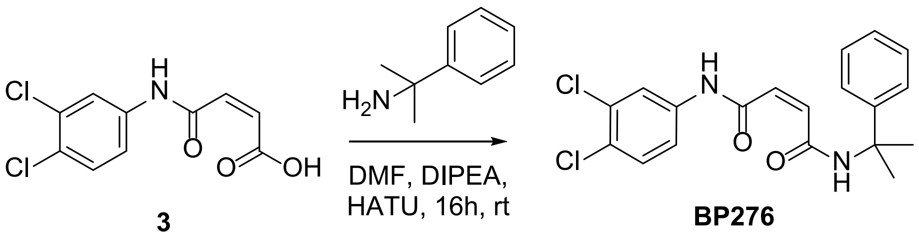

Scheme 32: Synthesis of **BP276**.

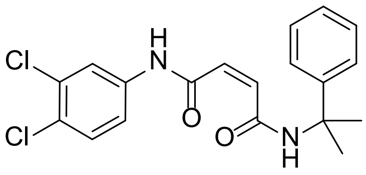

BP276. *N*[1]-(3,4-dichlorophenyl)-*N*[4]-(2-phenylpropan-2-yl)maleamide was prepared as outlined in General Procedure D using 2-phenylpropan-2-amine on a 0.412mmol scale (13.8mg, 9% yield). ^{1}H NMR (400 MHz, $CDCl_3$) δ 11.98 (s, 1H), 7.82 (d, *J* = 2.3 Hz, 1H), 7.51 – 7.45 (m, 1H), 7.41 – 7.27 (m, 7H), 6.16 (q, *J* = 13.4 Hz, 2H), 1.76 (s, 6H). ^{13}C NMR (101 MHz, $CDCl_3$) δ 164.67, 162.66, 145.70, 137.66, 135.91, 132.52, 131.42, 130.31, 128.55, 127.07, 124.72, 121.82, 119.57, 77.36, 77.04, 76.72, 57.03, 28.78. ^{13}C NMR (101 MHz, $CDCl_3$) δ 164.67, 162.66, 145.70, 137.66, 135.91, 132.52, 131.42, 130.31, 128.55, 127.07, 124.72, 121.82, 119.57, 77.36, 77.04, 76.72, 57.03, 28.78. HPLC-MS t_R = 9.791min, 95.2% purity, [$C_{19}H_{18}Cl_2N_2O_2$ - H] = 375.1, found 375.1.

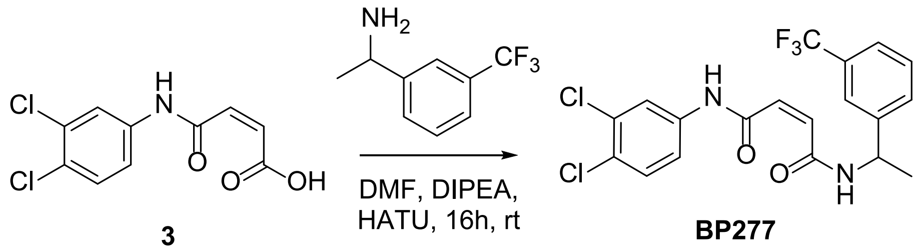

Scheme 33: Synthesis of **BP277**.

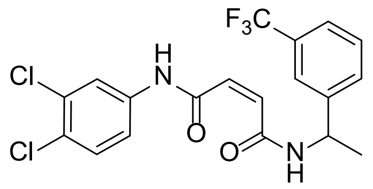

BP277. *N*[1]-(3,4-dichlorophenyl)-*N*[4]-(1-(3-(trifluoromethyl)phenyl)ethyl)maleamide was prepared as outlined in General Procedure D using 1-(3-(trifluoromethyl)phenyl)ethan-1-amine on a 0.581mmol scale (101.7mg, 41% yield). ^{1}H NMR (400 MHz, DMSO) δ 10.89 (s, 1H), 8.96 (d, *J* = 7.8 Hz, 1H), 8.03 (d, *J* = 2.4 Hz, 1H), 7.75 (s, 1H), 7.65 (d, *J* = 7.4 Hz, 1H), 7.62 – 7.53 (m, 3H), 7.46 (dd, *J* = 8.7, 2.4 Hz, 1H), 6.35 (s, 2H), 5.06 (p, *J* = 7.2 Hz, 1H), 1.40 (d, *J* = 7.0 Hz, 3H). ^{13}C NMR (101 MHz, DMSO) δ 163.99, 163.96, 145.70, 138.98, 131.48, 131.38, 131.02, 130.63, 130.34, 129.27, 124.82, 123.51, 123.47, 122.67, 122.63, 120.37, 119.24, 47.76, 40.15, 39.94, 39.73, 39.52, 39.31, 39.10, 38.89, 22.22. HPLC-MS t_R = 9.716min, 96.9% purity, [$C_{19}H_{15}Cl_2F_3N_2O_2$ - H] = 429.1, found 429.0.

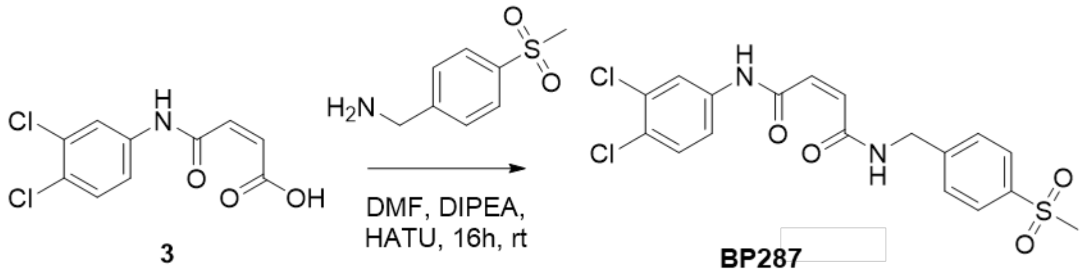

Scheme 34: Synthesis of **BP287**.

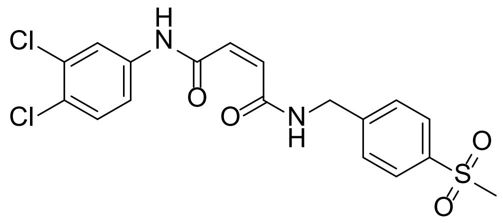

BP287. *N*[1]-(4-methylsulfonyl)benzyl-*N*[4]-(3,4-dichlorophenyl)maleamide was prepared as outlined in General Procedure D using 4-methylsulfonyl benzylamine on a 0.576mmol scale (72.5mg, 29% yield). ^{1}H NMR (400 MHz, $CDCl_3$) δ 12.03 (s, 1H), 8.11 (s, 1H), 7.88 (dd, J = 5.7, 2.8 Hz, 3H), 7.55 (d, J = 8.0 Hz, 2H), 7.48 (dd, J = 8.8, 2.4 Hz, 1H), 7.36 (d, J = 8.7 Hz, 1H), 6.19 (s, 2H), 5.22 (p, J = 7.1 Hz, 1H), 3.05 (s, 3H), 1.58 (d, J = 7.0 Hz, 3H). ^{13}C NMR (101 MHz, $CDCl_3$) δ 164.82, 162.78, 149.19, 139.57, 137.64, 136.04, 132.76, 130.95, 130.59, 127.97, 127.46, 121.93, 119.65, 77.48, 77.16, 76.84, 49.43, 44.61, 38.80, 21.73. HPLC-MS t_R = 8.789min, 93.5% purity, [$C_{19}H_{18}Cl_2N_2O_4S$ - H] = 439.0, found 439.0.

Appendix References

Dolomanov OV, Bourhis LJ, Gildea RJ, Howard JAK, Puschmann H, Olex2: A complete structure solution, refinement and analysis program, *J. Appl. Cryst.*, (2009), **42**, 339-341.

Domaszewska, Teresa, Lisa Scheuermann, Karin Hahnke, Hans Mollenkopf, Anca Dorhoi, Stefan HE Kaufmann, and January Weiner 3rd. 2017. “Concordant and Discordant Gene Expression Patterns in Mouse Strains Identify Best-Fit Animal Model for Human Tuberculosis.” *Scientific Reports* 7 (1): 12094.

Sheldrick, G.M., XT, *Acta Cryst.*, (2015), **A71**, 3-8.

Sheldrick, G.M., XL, *Acta Cryst.*, (2015), **C71**, 3-8.

Software for the Integration of CCD Detector System Bruker Analytical X-ray Systems, Bruker AXS, Madison, WI (after 2013).
