# Supplementary figures and images for: Disrupting TSLP–TSLP receptor interactions via putative small molecule inhibitors yields a novel and efficient treatment option for atopic diseases
Source: EMBO Mol Med. 2024 Jun 14;16(7):9. doi: 10.1038/s44321-024-00085-3 (PMC11250841; doi:10.1038/s44321-024-00085-3)

Fig. 3G

Western blot: Primary CD4+ T cells

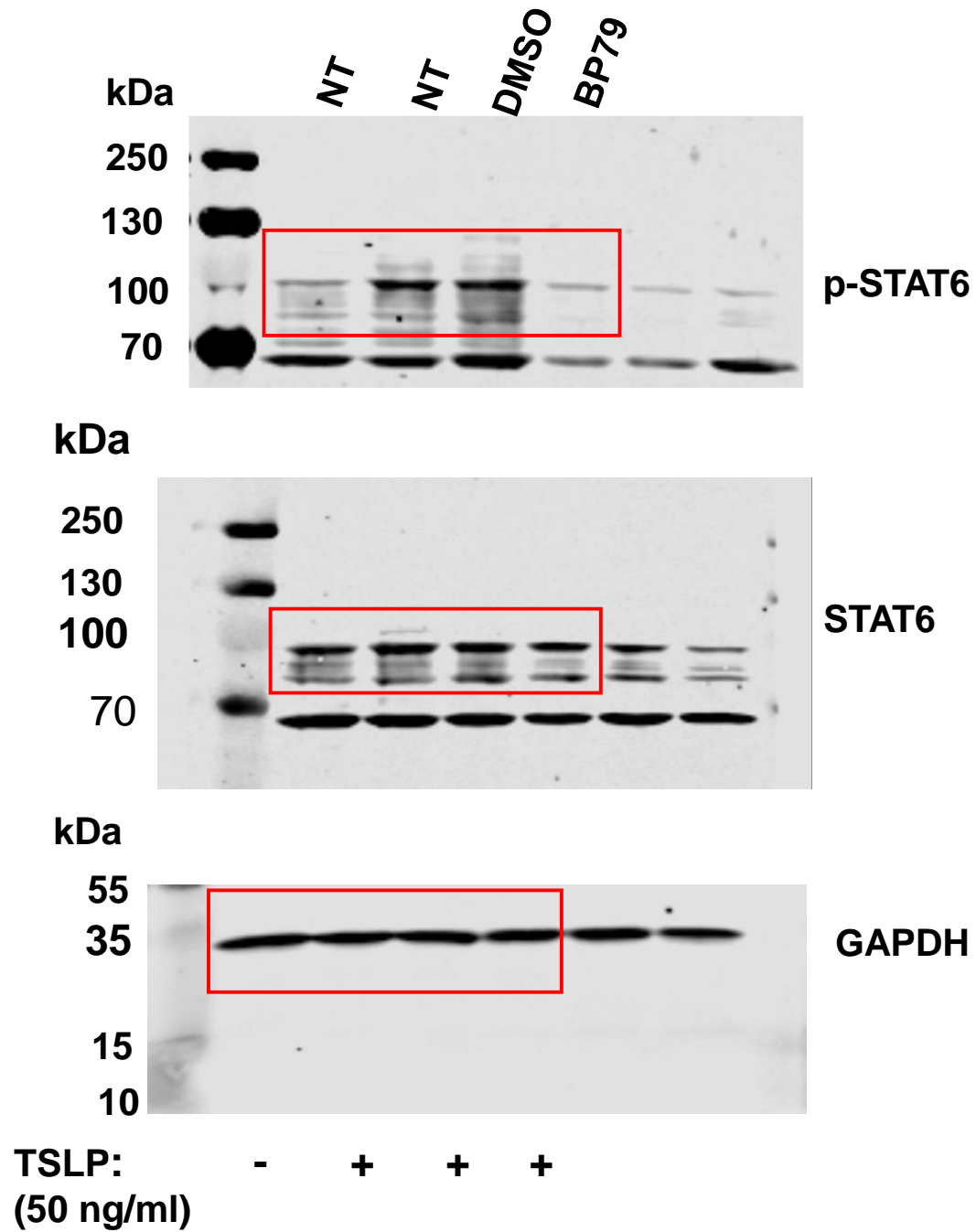

Supplement: Supplementary file 5 — Source data Fig. 3 [file 44321_2024_85_MOESM5_ESM.zip › Figure 3/Fig. 3G/Fig. 3G.pdf]

Fig. 3H

Western blot: Primary Keratinocyte cells

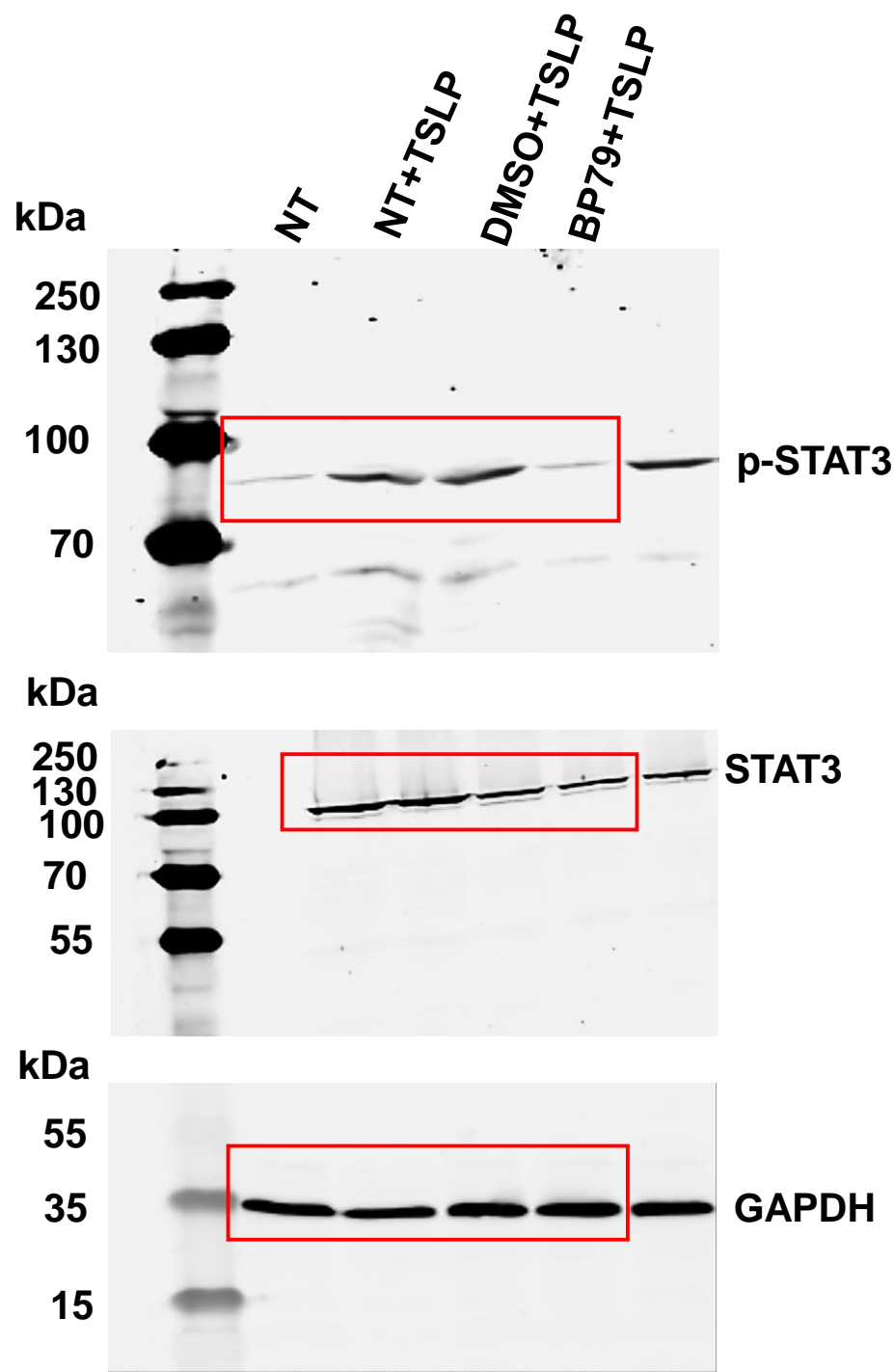

Supplement: Supplementary file 5 — Source data Fig. 3 [file 44321_2024_85_MOESM5_ESM.zip › Figure 3/Fig. 3H/Fig. 3H.pdf]

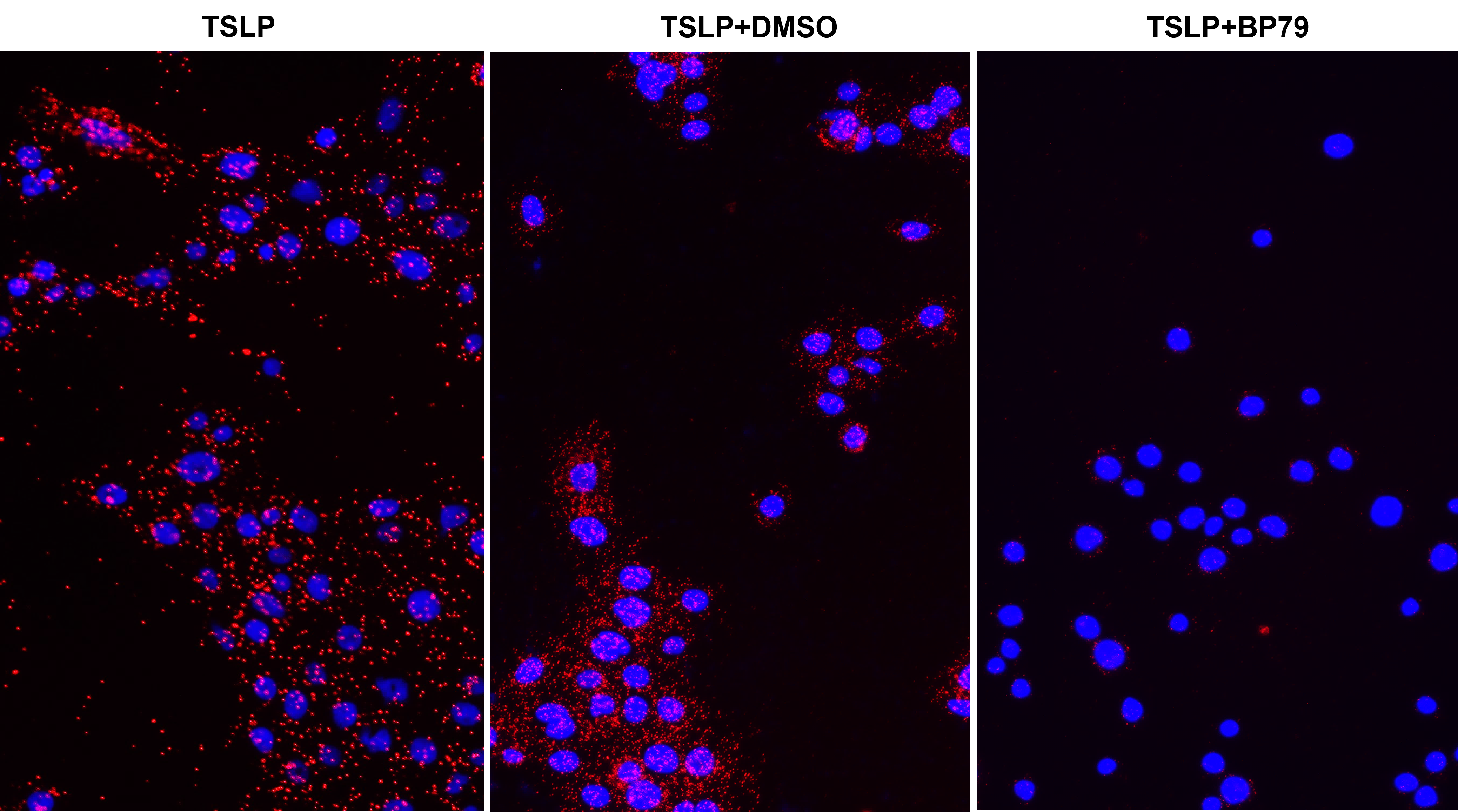

Supplement: Supplementary file 6 — Source data Fig. 4 [file 44321_2024_85_MOESM6_ESM.zip › Figure 4/Fig. 4A/Fig. 4A_PLA_Image.tif]

***In vitro* Thermal Shift Assay:**

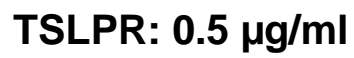

Supplement: Supplementary file 6 — Source data Fig. 4 [file 44321_2024_85_MOESM6_ESM.zip › Figure 4/Fig. 4B/Fig. 4B.pdf]

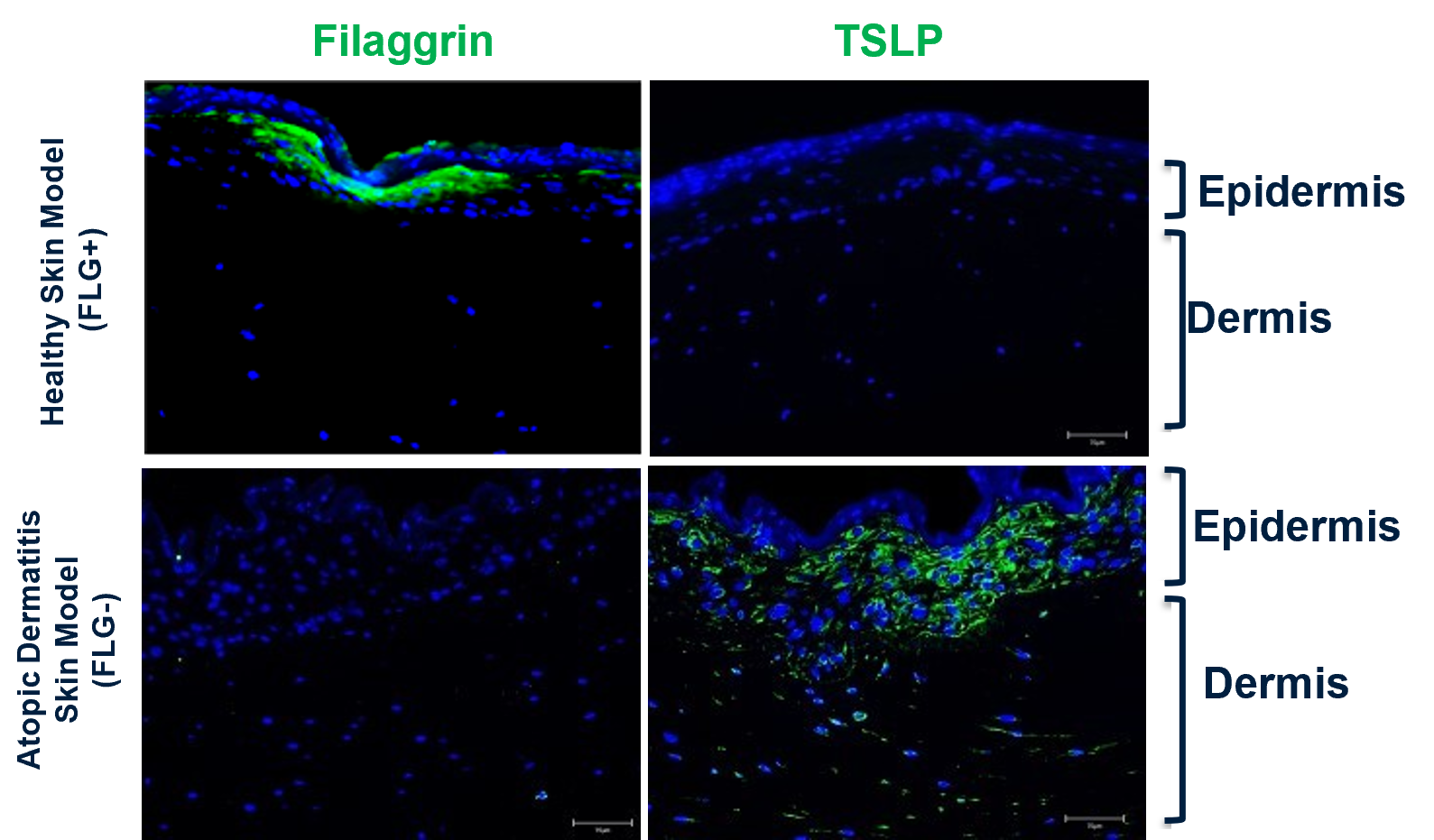

Supplement: Supplementary file 7 — Source data Fig. 5 [file 44321_2024_85_MOESM7_ESM.zip › Figure 5/Fig. 5B/Fig. 5B.tif]

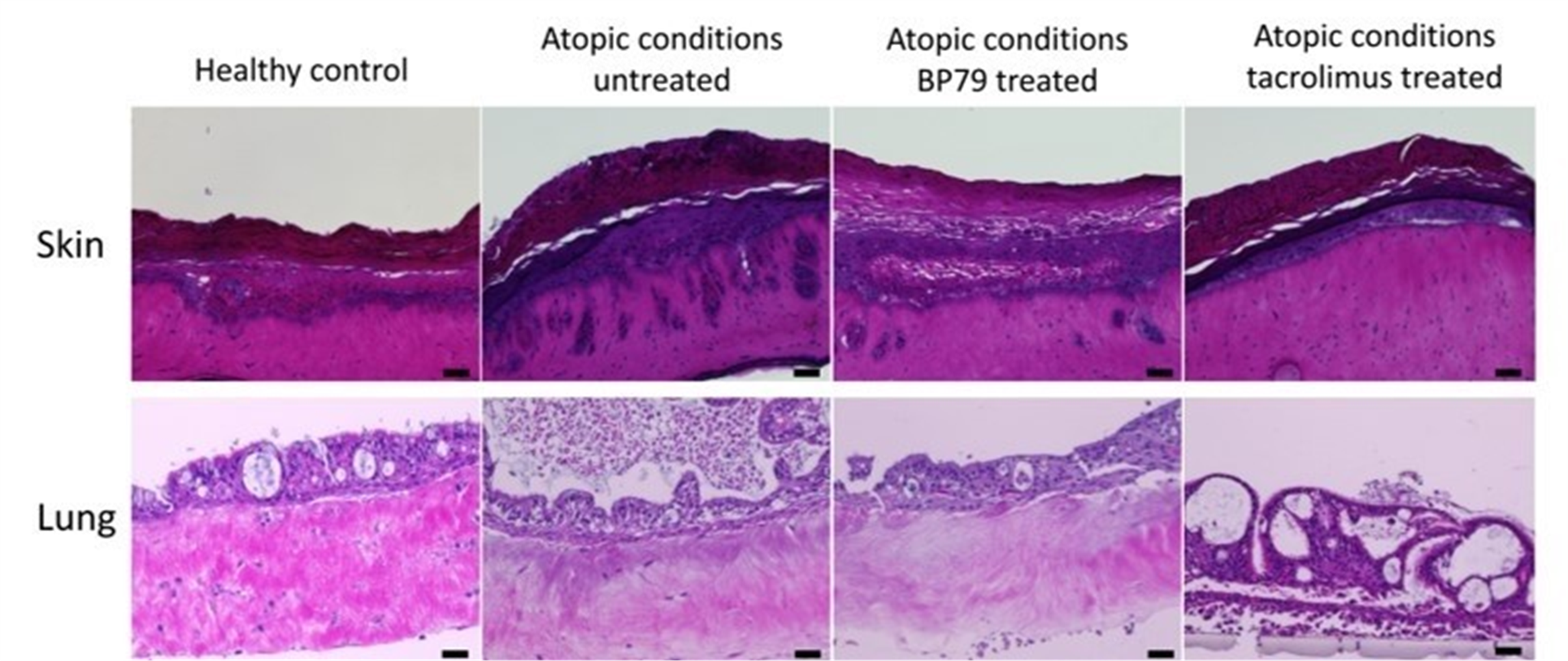

Supplement: Supplementary file 8 — Source data Fig. 6 [file 44321_2024_85_MOESM8_ESM.zip › Figure 6/Fig. 6C/Fig. 6C.tif]

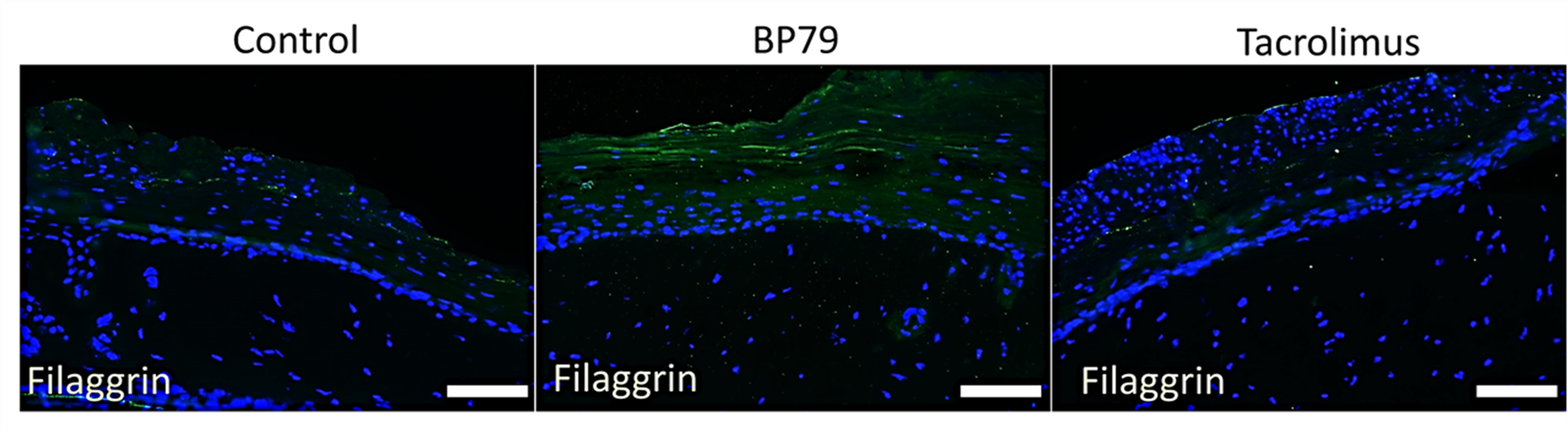

Supplement: Supplementary file 8 — Source data Fig. 6 [file 44321_2024_85_MOESM8_ESM.zip › Figure 6/Fig. 6F/Fig. 6F.tif]

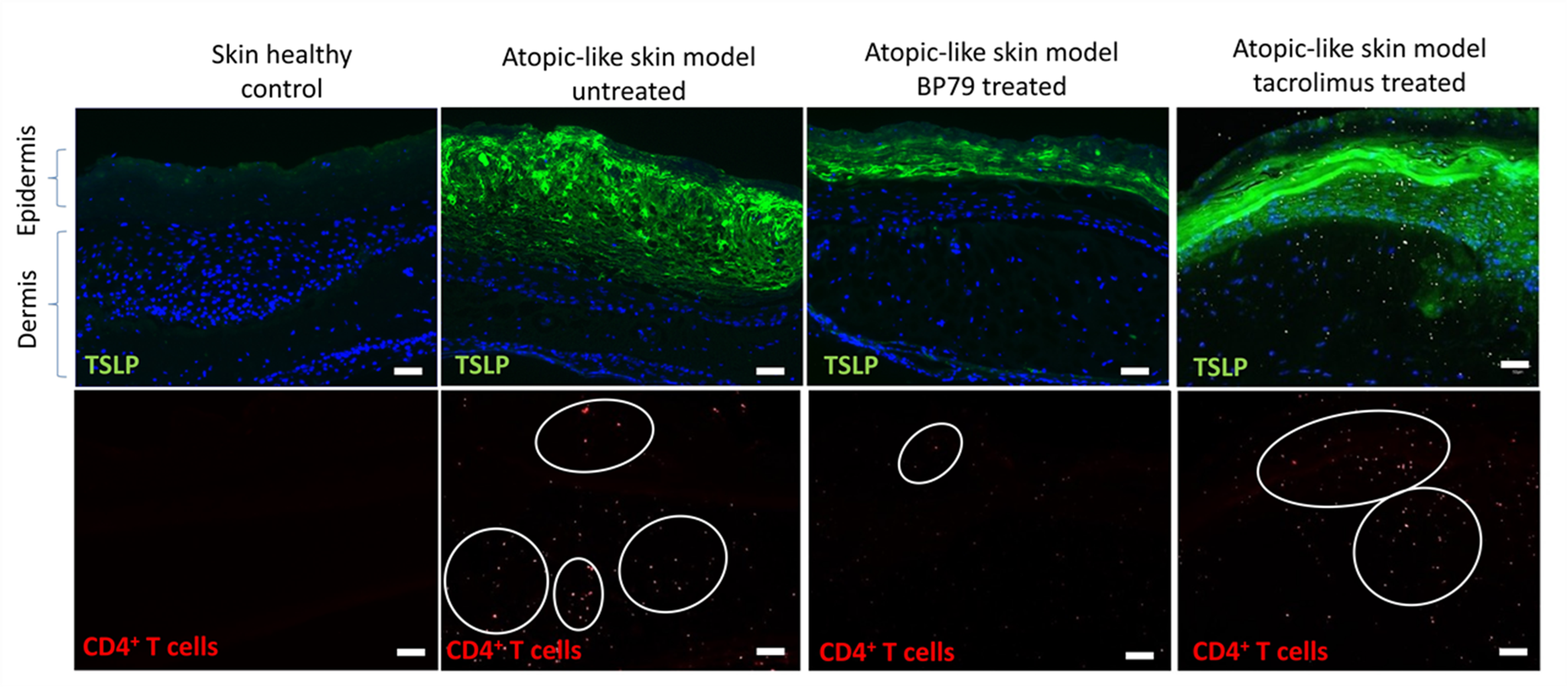

Supplement: Supplementary file 8 — Source data Fig. 6 [file 44321_2024_85_MOESM8_ESM.zip › Figure 6/Fig. 6G/Fig. 6G.tif]
