# Supplementary material for: Disrupting TSLP–TSLP receptor interactions via putative small molecule inhibitors yields a novel and efficient treatment option for atopic diseases
Source: EMBO Mol Med. 2024 Jun 14;16(7):9. doi: 10.1038/s44321-024-00085-3 (PMC11250841; doi:10.1038/s44321-024-00085-3)
Supplement: Supplementary file 9 — Expanded View Figures [file 44321_2024_85_MOESM9_ESM.pdf]

Expanded View Figures

Figure EV1. Biological screen of twenty-eight BP79 analogs.

(**A**) Chemical structures of BP79 analogs produced. (**B**) IL-13 expression in HuT78 cells after treatment with 1μm of inhibitor for 36 h ($n = 2$). (**C**) IL-4 expression in HuT78 cells after treatment with 1 μm of inhibitor for 36 h. Bars represent mean percentage cytokine expression relative to the DMSO control ($n = 2$). Non-treated (NT), PMA-ionomycin stimulated (PI), PMA-ionomycin and TSLP-stimulated (PI + TSLP), and a PMA-ionomycin + TSLP-stimulated 0.4% DMSO vehicle control (DMSO) were included. Data information: All data are presented as mean ± SEM. *n* represents the number of biological replicates. Statistical analysis was performed using Student's *t* test, $^{*}P \leq 0.05$, $^{**}P \leq 0.01$, $^{***}P \leq 0.001$.

© The Author(s)

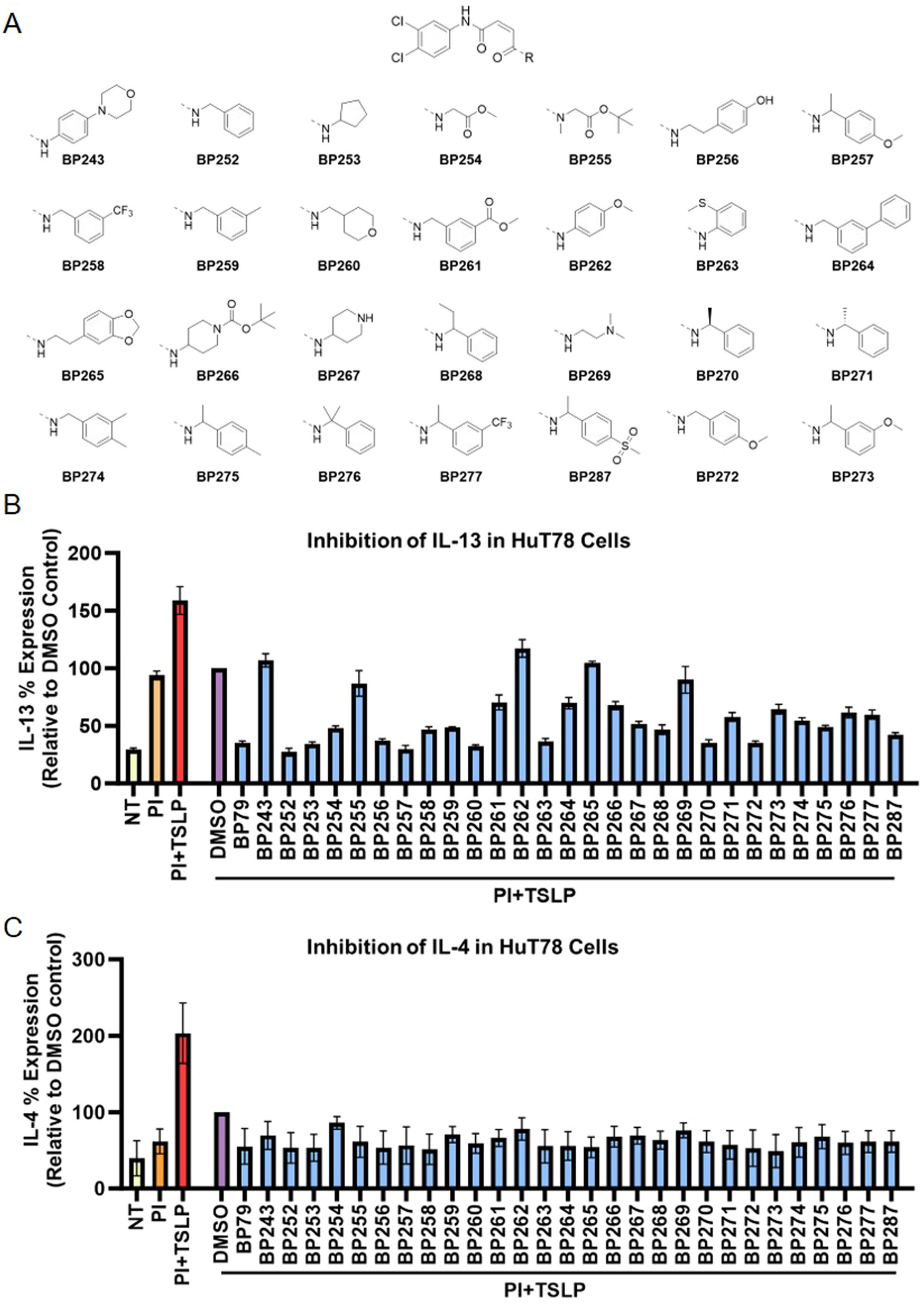
A
BP243
BP252
BP253
BP254
BP255
BP256
BP257
BP258
BP259
BP260
BP261
BP262
BP263
BP264
BP265
BP266
BP267
BP268
BP269
BP270
BP271
BP274
BP275
BP276
BP277
BP287
BP272
BP273
B
Inhibition of IL-13 in HuT78 Cells
IL-13 % Expression (Relative to DMSO Control)
0
50
100
150
200
NT
PI
PI+TSLP
DMSO
BP79
PI+TSLP
C
Inhibition of IL-4 in HuT78 Cells
IL-4 % Expression (Relative to DMSO control)
0
100
200
300
PI+TSLP

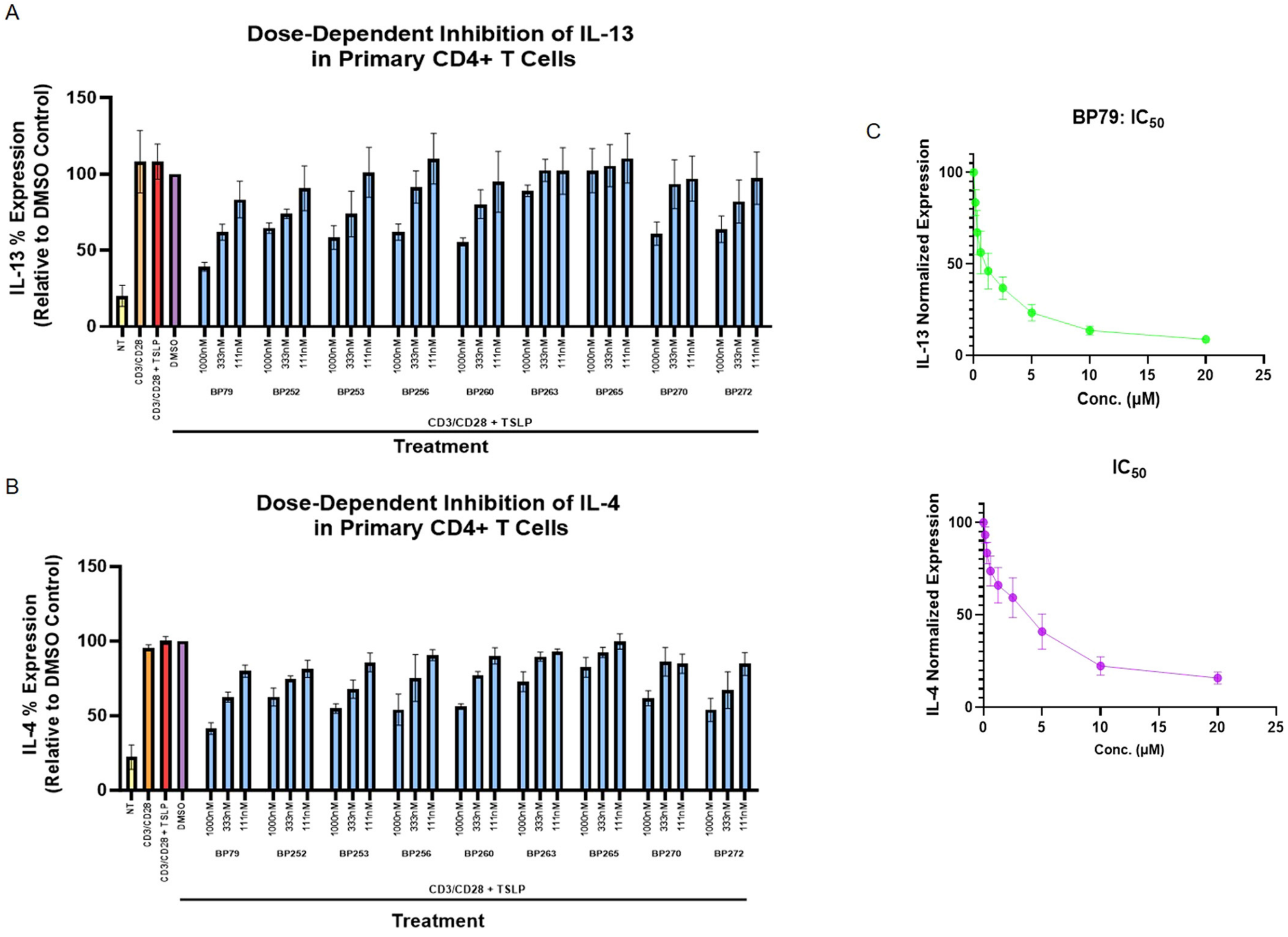

Figure EV2. Dose-dependent screen of BP79 analogs in CD4 + T cells.

(**A**) IL-13 expression in primary CD4 + T cells after treatment with 111 nM, 333 nM, and 1000 nM of inhibitor for 36 h. (**B**) IL-4 expression primary CD4 + T cells after treatment with 111 nM, 333 nM, and 1000 nM of inhibitor for 36 h. Bars represent mean percent cytokine expression relative to the DMSO control, $n = 3$. Non-treated (NT), CD3/CD28 stimulated (CD3/CD28), CD3/CD28 and TSLP-stimulated (CD3/CD28 + TSLP), and vehicle (CD3/CD28 + TSLP + DMSO) controls were included. (**C**) Dose–response curves of BP79, which were used to calculate the IC_{50} value and HillSlope ($n = 3$). Data information: All data are presented as mean ± SEM. *n* represents the number of biological replicates. Statistical analysis was performed using Student's *t* test, *$P \leq 0.05$, **$P \leq 0.01$, ***$P \leq 0.001$.

© The Author(s)

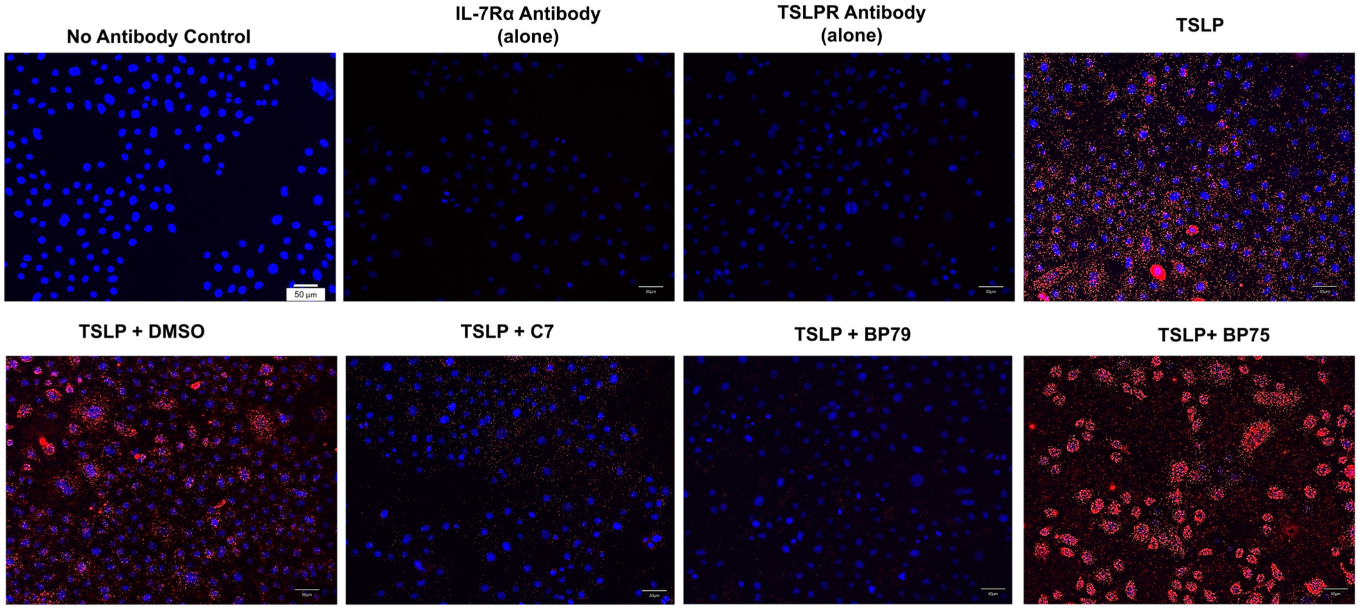

Figure EV3. Full panel images of proximity ligation assay (PLA).

PLA was performed to check the inhibition of TSLP-mediated ternary complex formation with BP79. Primary keratinocyte cells were treated with the TSLP inhibitors and stimulated with TSLP. Cells were fixed and the interaction between TSLPR and IL-7Rα was observed with PLA assay. C7 and BP79 inhibited the TSLP:TSLPR:IL-7Rα ternary complex formation. Scale bar = 50 μm.

© The Author(s)
